# Ferroelectric materials for neuroinspired computing applications

**DOI:** 10.1016/j.fmre.2023.04.013

**Published:** 2023-05-19

**Authors:** Dong Wang, Shenglan Hao, Brahim Dkhil, Bobo Tian, Chungang Duan

**Affiliations:** a Key Laboratory of Polar Materials and Devices (MOE), Ministry of Education, Shanghai Center of Brain-inspired Intelligent Materials and Devices, Department of Electronics, East China Normal University, Shanghai 200241, China; b Laboratoire Structures, Propriétés et Modélisation des Solides, CentraleSupélec, CNRS-UMR8580, Université Paris-Saclay, Paris 91190, France; c Zhejiang Lab, Hangzhou 310000, China; d Collaborative Innovation Center of Extreme Optics, Shanxi University, Shanxi 030006, China

**Keywords:** Ferroelectric materials, Ferroelectric synaptic devices, Artificial neural network, In-memory computing, In-sensor computing

## Abstract

In recent years, the emergence of numerous applications of artificial intelligence (AI) has sparked a new technological revolution. These applications include facial recognition, autonomous driving, intelligent robotics, and image restoration. However, the data processing and storage procedures in the conventional von Neumann architecture are discrete, which leads to the “memory wall” problem. As a result, such architecture is incompatible with AI requirements for efficient and sustainable processing. Exploring new computing architectures and material bases is therefore imperative. Inspired by neurobiological systems, in-memory and in-sensor computing techniques provide a new means of overcoming the limitations inherent in the von Neumann architecture. The basis of neural morphological computation is a crossbar array of high-density, high-efficiency non-volatile memory devices. Among the numerous candidate memory devices, ferroelectric memory devices with non-volatile polarization states, low power consumption and strong endurance are expected to be ideal candidates for neuromorphic computing. Further research on the complementary metal–oxide–semiconductor (CMOS) compatibility for these devices is underway and has yielded favorable results. Herein, we first introduce the development of ferroelectric materials as well as their mechanisms of polarization reversal and detail the applications of ferroelectric synaptic devices in artificial neural networks. Subsequently, we introduce the latest developments in ferroelectrics-based in-memory and in-sensor computing. Finally, we review recent works on hafnium-based ferroelectric memory devices with CMOS process compatibility and give a perspective for future developments.

## Graphical abstract

**Figure:**
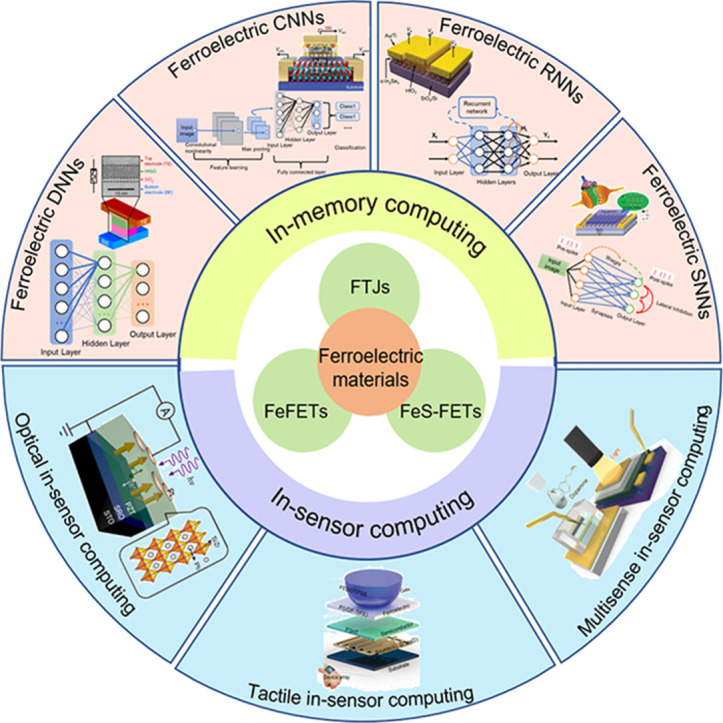

## 1 Introduction

Conventional computers are built on the basis of complementary metal–oxide–semiconductor (CMOS) logic and the von Neumann architecture, which have discrete data storage and processing and serial transmission of instructions and data access [1,2]. These characteristics severely limit the running speed of computers during data flow, (i.e., memory access) and engenders high energy consumption. Moreover, the “memory wall” problem is intensified during the execution of intelligence tasks such as image recognition and semantic understanding. Conventional computers can no longer meet the needs of the smart society. Researchers and innovators are therefore addressing these obstacles from multiple perspectives, from basic devices to system architectures, with the aim of developing a new type of computing system that integrates storage and processing and is known as in-memory computing. In this new technology, massively parallel and energy-efficient neural morphological computing could be implemented through high-density cross arrays containing devices with non-volatile memory. Directly using memory for data processing or computing overcomes the limitations engendered by the memory wall problem. Although in-memory computing was first proposed by Kautz et al. [3] as early as 1969, experimental research on non-volatile memory devices for in-memory computing started to bloom only recently. Efforts are ongoing to promote memory devices in the post-Moore era and thus better support in-memory computing technology for multilevel collaborative innovations such as new materials, device mechanisms, architectures, and integrated systems [4].

In addition to having the characteristics such as high speed, low cost, non-volatility, and long-term stability, an in-memory device should offer multilevel storage, threshold switching, and learning rules such as spike-time-dependent plasticity (STDP) or spike-rate-dependent plasticity (SRDP) [5]. With the rediscovery of memristors in 2008 [6], numerous memristor-based in-memory computing strategies have been proposed for enhancing energy efficiency and computational speed. Thus, memristors can address the inefficiency problem engendered by frequent information scheduling in the traditional von Neumann architecture [7]. However, the development of in-memory computing is limited by the bottleneck created by the need for analog-to-digital and digital-to-analog conversion processes between information acquisition, transmission, and intelligent processing. Inspired by the visual system of the human brain, researchers are attempting to develop an in-sensor computing system that integrates sensing, memory, and computing. In in-sensor computing, the sensor of collecting programmable and multilevel analog signals, unit of data storage and computing are integrated together; this further improves the system's energy efficiency [8,9]. This technology has fostered a new area of technological growth for the development of this flourishing field of “in-memory computing”.

Of the various types of non-volatile memory used in in-memory and in-sensor computing, those based on ferroelectric materials have attracted considerable research interest owing to their low energy consumption, high speed, and strong fatigue resistance [10]. The non-volatile spontaneous polarization state of ferroelectric thin films can be reversed by applying an electric field [11]. During ferroelectric polarization reversal, the states of the ferroelectric domains can be continuously changed by manipulating the amplitude of the applied electric field and the duration for which it is applied. Moreover, such abundant intermediate polarization states are non-volatile [12]. The continuous change in ferroelectric polarization due to external field modulation is highly like the continuous change in the weights of connections between biological synapses, which is referred to as synaptic plasticity. The correspondence between ferroelectric polarization plasticity and biological synaptic plasticity can enable the development of novel artificial ferroelectric synaptic devices with low power consumption, high stability, high repeatability, and high controllability [13,14]. In addition, ferroelectric materials can be coupled with various external stimuli through effects such as optoelectronic, piezoelectric, and pyroelectric effects [15,16]. Accordingly, ferroelectric synaptic components constitute new physical building blocks for creating intelligent systems based on the in-sensor computing framework.

In this paper, we first briefly introduce the properties of ferroelectrics and the synaptic devices based on ferroelectric materials. Subsequently, we summarize and discuss the main research advances in ferroelectric-based neuromorphic computing, including in-memory and in-sensor computing.

## 2 Ferroelectric-material-based synaptic devices

Having been discovered a century ago, ferroelectric materials exhibit several useful functions that already leveraged in electromechanical transducers, electro-optical modulators, and thermal sensors [17]. Ferroelectrics also have promising applications in non-volatile memory and neuromorphic devices. The switching of ferroelectric polarization consumes little energy because it is driven by an electric field (i.e., voltage), and the switched polarization state remains stable when the field is withdrawn; that is, the state is non-volatile. The process of polarization reversal is ultrafast, occurring within tens of nanoseconds. Thus, ferroelectric materials offer major advantages for memory and synaptic device applications in neuromorphic computing [18]. This section briefly introduces ferroelectric materials and the polarization reversal mechanism, and it also provides a detailed discussion of memory devices based on ferroelectric materials.

### 2.1 Ferroelectric materials

**Fig. 1:**
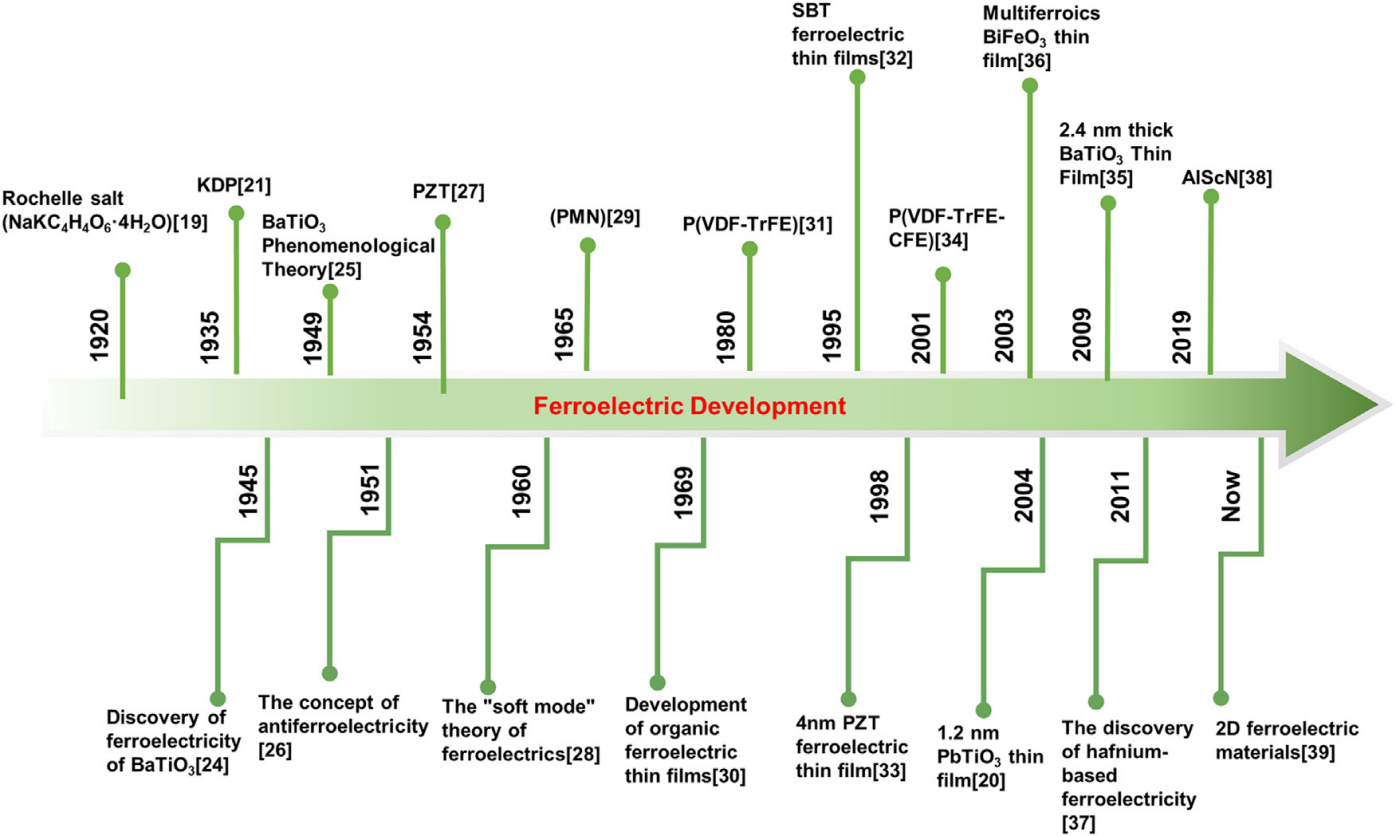
**History of the development of ferroelectric**.

Valasek [19] was the first to report the polarization hysteresis effect of Rochelle salt (NaKC_4_H_4_O_6_·4H_2_O) in 1920, and detailed the first characteristic hysteresis ferroelectric loop between charge and the applied electric field. Research on ferroelectric materials has since been boosted by the discovery of remarkable materials such as KH_2_PO_4_, BaTiO_3_, KNbO_3_, KTaO_3_, LiNbO_3_, PbTiO_3,_ (Pb,Zr)TiO_3_ (PZT)_,_ PbMg_1/3_Nb_2/3_O_3_, KNaNbO_3_, SrBi_2_Ta_2_O_9_, BiFeO_3_, and some organic materials including polyvinylidene fluoride (PVDF), the copolymer of poly(vinylidene fluoride-trifluoro-ethylene) [P(VDF-TrFE)], and terpolymers [20], [21], [22], [23], [24], [25], [26], [27], [28], [29], [30], [31], [32], [33], [34], [35], [36]. Recently, hafnium-based systems (HfO_2_) [37], aluminum scandium nitrogen (AlScN) [38], and two-dimensional (2D) ferroelectric materials [39] have become new ferroelectric materials of interest owing to their promising potential use in memory and computing applications. Fig. 1 illustrates a summary of the development history of ferroelectric materials. Research on ferroelectric materials has achieved considerable progress in theory and modeling, material synthesis and processing, multiscale and time-dependent characterizations, and applications. Ferroelectric materials usually exhibit piezoelectric effects, pyroelectric and electrocaloric responses, electro-optic and nonlinear optical activities, and other properties. Therefore, they have an essential function in many devices such as capacitors, piezoelectric transducers, non-volatile memory devices, medical ultrasound imaging equipment, and spatial light modulators. In recent decades, improvements in preparation technology have led to a series of breakthroughs in more varied research fields related to ferroelectric materials, such as ferroelectric tunnel junctions (FTJs) [40,41], ferroelectric field-effect transistors (FeFETs) [42,43], photovoltaic devices [44], and flexible devices [45]. Ferroelectric materials have also been found to have extensive application prospects in terms of home appliances, communications, national defense, and aerospace; they are thus currently one of the frontiers and hot spots of high-tech research.

**Fig. 2:**
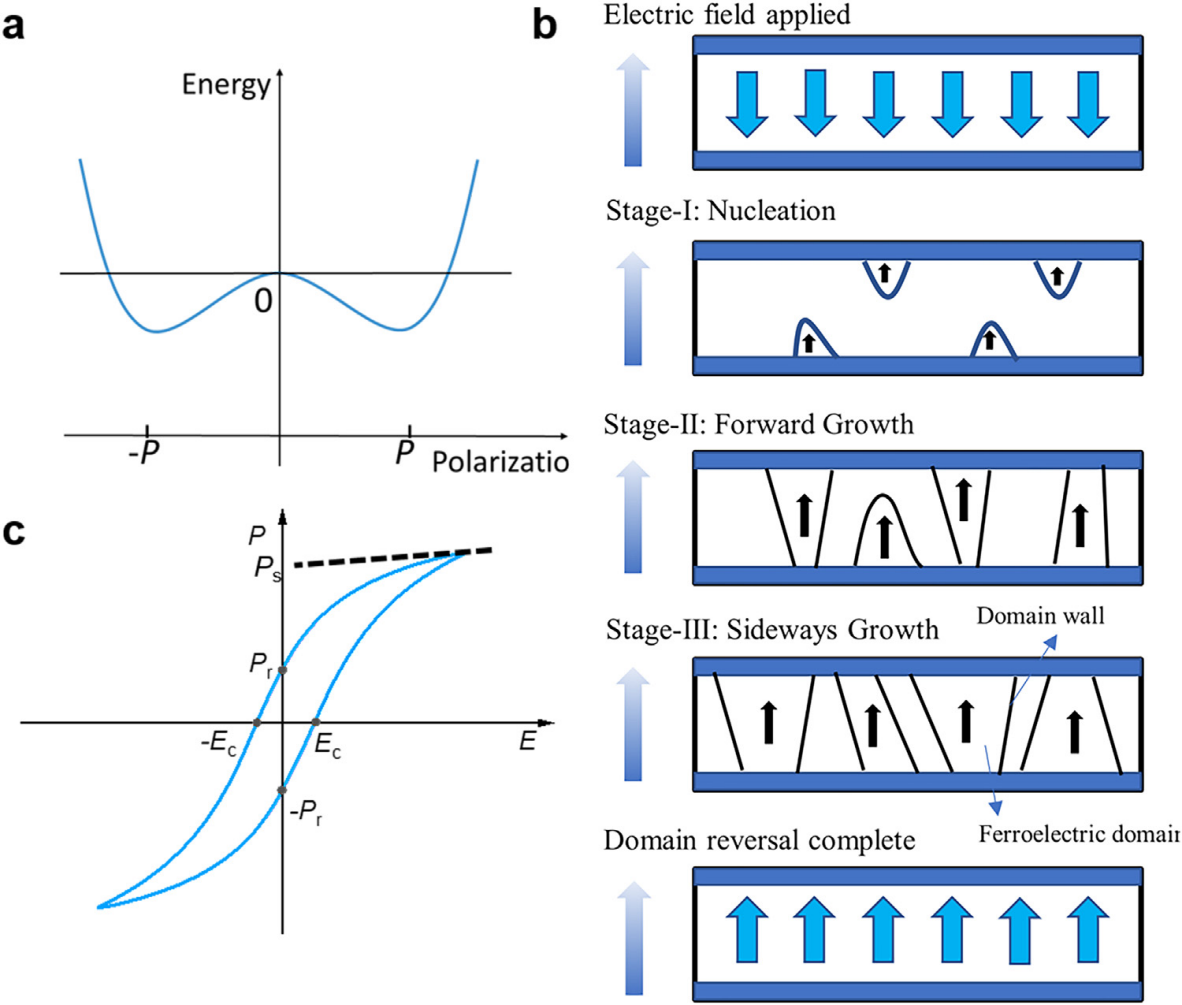
**Ferroelectrics characteristics.** (a) Energy landscape of ferroelectric phases; (b) main process of polarization reversal [46] (Copyright 2005 American Physical Society); and (c) hysteresis loop.

In a ferroelectric material, the space-reversal symmetry is broken; therefore, the centers of positive and negative ionic charges in the unit cell do not overlap, and this creates a spontaneous electric dipole moment [17]. All dipoles are oriented in the same direction in each domain, and two adjacent domains are separated by a boundary called domain wall. Ferroelectric polarization reversal refers to a phenomenon in which the orientation of the spontaneous polarization of a ferroelectric domain change due to an external electric field. This is the most basic ferroelectric property and is associated with a bistable switching process (polarization up and polarization down), as displayed in Fig. 2a. It is also the physical principle that enables the realization of binary ferroelectric storage. The down- and up-polarization states represent “0” and “1” memory states, respectively. Fig. 2b presents the polarization reversal mechanism that involves the formation of new domains with the movement of domain walls. This mechanism comprises four main stages. Consider a material in which most of the domains have already been oriented downwards, forming a single domain as shown in Fig. 2b; this corresponds to −P_remanent_ (−*P_r_*) in the hysteresis loop in Fig. 2c. When an upward-oriented electric field is applied, some domains with up-polarization nucleate (stage I). As the electric field becomes stronger, the new domains grow in the vertical direction until they reach the bottom electrode (stage II). Subsequently, the domains grow laterally through the movement of the domain walls (stage III) until these upward domains merge at the expense of the initial downward-oriented domains [46].

It presents the polarization–electric field (*P–E)* hysteresis loop in Fig. 2c. As the voltage increases, ferroelectric domains with opposite orientation to the electric field are reversed. When half of the domains are reversed, the total polarization is null and the electric field corresponds to the coercive electric field *E*_c_. The domain reversal progresses until all the domains are reversed. As the strength of the electric field increases, the total polarization continues to increase until it reaches the saturation polarization *P*_s_. When the voltage is gradually reduced, some of the domains deviate from the polarization direction under the action of intra-lattice stress, and this results in a slight decrease in the polarization strength. However, most of the domains retain the same polarization direction as the electric field direction, so that a certain polarization is retained when the voltage is reduced to zero; this is the remanent polarization *P*_r_. The degree of opening of the hysteresis loop depends on the strength of the applied electric field (sufficient to saturate the polarization) and the frequency of the field that is related to the dynamic of the ferroelectric domains [47].

Numerous studies have demonstrated that in ferroelectric thin films, polarization reversal is accomplished by a creeping process in the domain walls within the appropriate strength range of the applied electric field above the coercive voltage [46]. The continuous state change that occurs during the ferroelectric polarization reversal process is closely related to the amplitude and duration time of the applied electric field. Notably, the intermediate polarization states are non-volatile. The continuous change in ferroelectric polarization with external field modulation is analogous to the continuous change in the weights of biological synapses. Accordingly, ferroelectric polarization plasticity can be considered to correspond to biological synaptic plasticity, indicating that novel artificial ferroelectric synaptic devices can be realized [18,48].

### 2.2 Memory devices based on ferroelectrics

Because of their multiple, non-volatile and tunable polarization states, ferroelectric materials have been extensively investigated for in-memory computing. The typical ferroelectric memory configurations are ferroelectric random-access memory (FeRAM), FTJs, FeFETs, and ferroelectric semiconductor field-effect transistors (FeS-FETs), which are expected to overcome the von Neumann bottleneck problem in information processing and storage. FeRAM structure consists of a transistor and capacitor, and the stored information is oriented in the direction of the polarization of the ferroelectric capacitor. FTJs are composed of an ultrathin ferroelectric tunnel barrier between two metallic electrodes or semiconductors in which the tunneling conductance can be manipulated by adjusting the polarization direction. FeFETs are used for in-memory computing thanks to the multiple states of their channel conductance; these states are controlled by the adjacent ferroelectric layers. In FeS-FETs, a ferroelectric semiconductor channel replaces the gate dielectric layer and channel layer that are found in conventional FeFETs; this combines both bound and movable charges, resulting in improved retention [10,16,49].

**Fig. 3:**
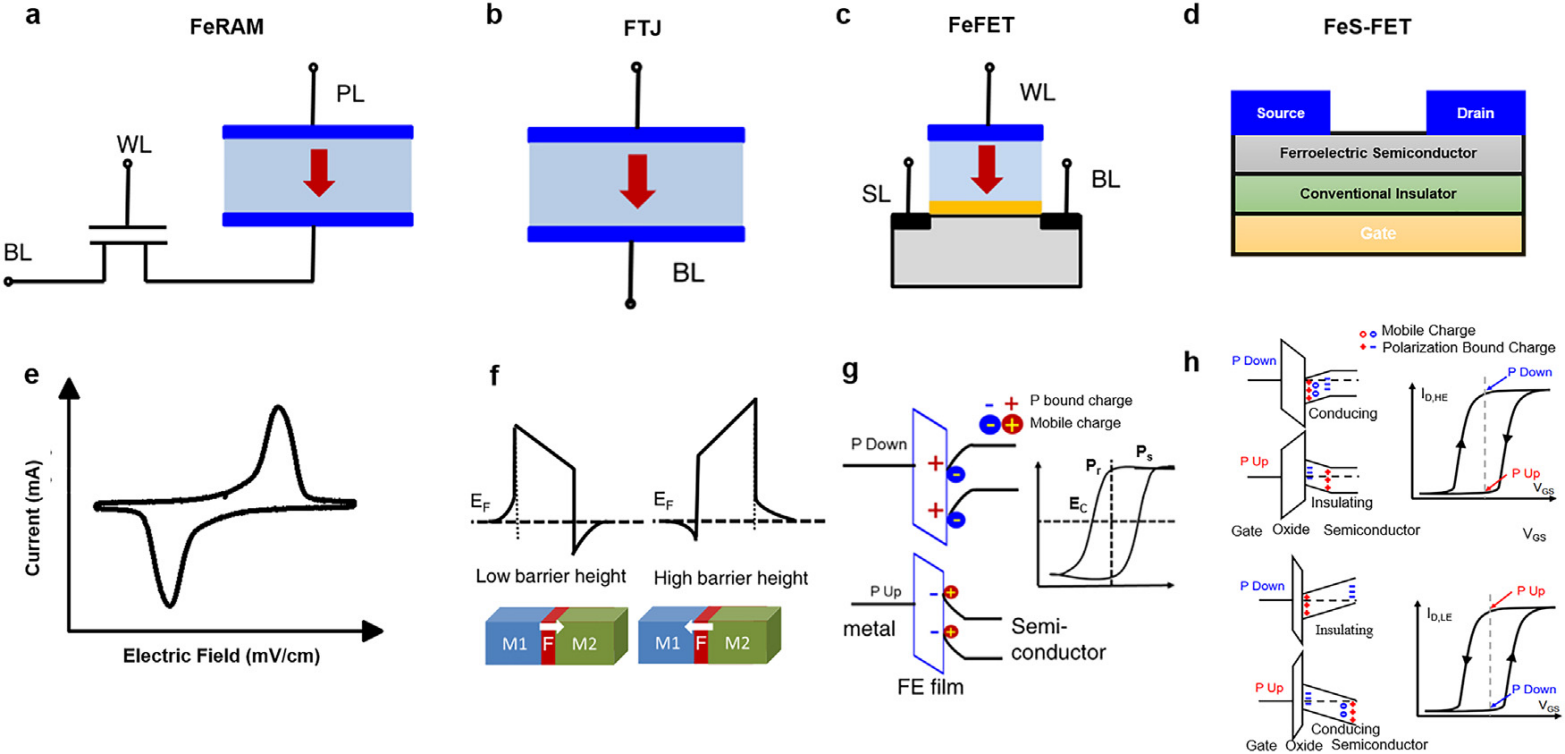
**Schematic of four ferroelectric memory devices.** (a) 1T1C FeRAM cell. (b) FTJ structure consisting of two metal/semiconductor electrodes and an ultrathin ferroelectric film; the resistance switching originates from the change in the direction of the ferroelectric layer's polarization. (c) FeFET cell, in which information is stored by modulating the polarization state of the ferroelectric layer [10] (Copyright 2021 Wiley-VCH GmbH). (d) FeS-FET; ferroelectric semiconductors are used as channels, and the gate insulators are traditional dielectrics. (e) Current–electric field curves of ferroelectric thin film. (f–h) Schematics of the corresponding device band diagrams. (f, g) [10] (Copyright 2021 Wiley-VCH GmbH.) (h) [62] (Copyright 2019 Springer Nature).

Conventional FeRAM (Fig. 3a) is similar to a dynamic random-access memory (DRAM) storage unit, comprising a transistor and capacitor structure (denoted 1T1C). Information can be stored as polarization and read through polarization switching **(**Fig. 3e). FeRAM based on conventional inorganic ferroelectric materials is limited by the necessity of considerable thickness for achieving robust ferroelectricity; moreover, the necessity of a large area for reading charges engenders difficulties in achieving high-density integration [10]. The destructive readout mode of charge reversal in FeRAM is also a limiting factor. However, as the advent of hafnium-based ferroelectric materials, coupled with back-end-of-line (BEOL) compatibility [50], low-voltage operation [51], and nanosecond switching speed [52], the scaling limit of 130-nm-wide nodes is expected to be broken; this will require the use of a three-dimensional (3D) structure (with trenches or stacks) to enhance the effective area of the storage capacitors to achieve this goal [53]. Some scholars have recently successfully demonstrated the integration of hafnia-based capacitors into FeRAM arrays. For example, Okuno et al. [54] reported a new 64-kbit 1T1C-FeRAM array based on Hf_0.5_Zr_0.5_O_2_ (HZO) with high durability and low-voltage operation. Maher et al. [55] conducted the research on the retention and endurance characteristics of HZO-based 3D-FeRAM cells. These efforts laid the foundation for 3D-FeRAM to overcome the storage bottleneck and be commercialized. However, for 3D-FeRAM to develop further, many theoretical and experimental breakthroughs remain to be made. Despite its commercial use, FeRAM with a destructive readout mode is not suitable for direct in-memory computing. Of the types of ferroelectric memory used in neuromorphic computing, the other three memory structures (i.e., FTJs, FeFETs, and FeS-FETs) remain the most promising. Accordingly, this section focuses on the potential of FTJs, FeFETs, and FeS-FETs as synapses for in-memory computing applications.

#### 2.2.1 Ferroelectric tunnel junctions

An FTJ (Fig. 3b) has a sandwich structure consisting of two metal or semiconductor electrodes and a very thin (typically only nanometers thick) ferroelectric film [10,49]. The effective barrier height of electron tunneling can be manipulated by adjusting the polarization state of the ferroelectric film. As illustrated in Fig. 3f, the tunneling conductance through the junction is changed to achieve a high- or low-resistance state (i.e., *R*_on_ or *R*_off_). This is the tunneling electroresistance (TER) effect [40]. A strong TER effect is required for memory devices in order to minimize errors and power consumption during reading or writing. FTJs were first reported in 1971 by Esaki et al. [56]. However, it was not until the 2000s that the FTJ was first demonstrated experimentally by using ferroelectric BaTiO_3_ [57].

First, Chanthbouala et al. [41] reported FTJ-based solid-state memories with the structure Au/Co/BaTiO_3_/La_0.67_Sr_0.33_MnO_3_, but the TER ratio was 100 at room temperature. Subsequently, Wen et al. [58] enhanced the TER effect by two orders of magnitude by using a Nb-doped SrTiO_3_ electrode. In addition to conducting research on perovskite-based ferroelectrics such as BiFeO_3_ or PbZr_0.2_Ti_0.8_O_3_, scholars have reported [49,59,60] further enhancement of the TER response in HfO_2_-based films, organic polymers (i.e., PVDF), and some van der Waals ferroelectrics such as CuInP_2_S_6_. A TER effect of >10^7^ was achieved by using a thin layer of CuInP_2_S_6_ as the ferroelectric barrier and placing it in contact with graphene [61]. Because of the modulation of polarization states in the ferroelectric layer, the aforementioned materials have promise as ferroelectric synapses for neuroinspired computing.

#### 2.2.2 FeFETs and FeS-FETs

FeFETs are transistor (1T) memory devices in which ferroelectric capacitors are integrated into the gate stack of field-effect transistors. In an FeFET, a ferroelectric material replaces the gate dielectric layer in the MOSFET structure (Fig. 3c) [10]. Ferroelectric polarization modulation in the opposite direction controls the concentration of carriers in the channel such that they are in an accumulation state or a depletion state; thus, the drain current of the FeFET varies with the shift in the polarization-dependent threshold voltage *V*_T_. This enables nondestructive reading and writing operations (Fig. 3g) that are similar to those in flash memory. The memory window Δ*V*_T_ of an FeFET is primarily dominated by the coercive voltage *V*_C_ of the ferroelectric layer. *V*_C_ is directly proportional to the thickness of the ferroelectric film and the strength of the coercive electric field *E*_C_ [63]. With the continual miniaturization of devices, the overall thickness of the gate is becoming increasingly crucial. Therefore, to maintain the necessary memory window, the ferroelectric gate material in an FeFET must have a large *E*_C_. Furthermore, in an FeFET, the port between the gate and the source/drain or the direct source–drain port can be used as an input. Consider, for example, supervised learning in a spiking neural network (SNN), which is an artificial neuronal network in which a set of spikes are received as the input and a series of spikes are produced as the output; here, the channel acts as a bridge to connect the presynaptic neurons with the postsynaptic neurons. The channel conductance of the transistor can be regulated by applying voltage pulses to the gate. Three-terminal synaptic devices have certain advantages over two-terminal devices in that the synaptic weights can be controlled by directly feeding back the signal to the gate, thereby achieving parallel learning [18].

In FeFETs, the mechanisms such as charge trapping at the interfaces, poor charge compensation by the semiconductor channel, and gate leakage current limit their further development. These mechanisms are also the main reason for the drift in the threshold voltage *V*_T_ and destruction of the memory state in FeFETs [16,64]. To solve these problems, a novel transistor structure, the FeS-FET, has been reported [62]. In this structure (Fig. 3d), ferroelectric semiconductors, as channel materials, can store two non-volatile polarized states. Furthermore, unlike in FeFETs, the built-in electric field formed by movable charges effectively shields the depolarization field in the transistor. In FeS-FETs, polarization charges accumulate on both surfaces (upper and lower) of the ferroelectric semiconductors. Thus, the two surfaces of the ferroelectric semiconductors have a major effect on the transistor's drain current *I*_D_. Taking α-In_2_Se_3_ FeS-FET as an example, the electric field is poorly distributed inside the semiconductor owing to the presence of movable charges in the channel [62]. This nonuniform distribution in turn affects the polarization switching in α-In_2_Se_3_ and is also the main reason for the clockwise and counterclockwise hysteresis of FeS-FETs. Fig. 3h displays the *I*_D_–*V*_GS_ curves of devices with a large versus a small effective oxide thickness (EOT). The difference between the two devices is that the semiconductor electric field in the large-EOT device is weak and cannot penetrate the upper surface of the semiconductor, whereas the strong electric field in the small-EOT device can trigger full polarization switching in the ferroelectric semiconductors [62]. These unique properties mean that FeS-FETs have greater applicability in in-memory computing and are expected to be developed for practical applications.

#### 2.2.3 FTJs as synapses

As mentioned, the bistable resistive switching effect of FTJs can be applied to non-volatile memory with large *R*_on_*_/_R*_off_, high speed, and low-energy operation. Moreover, because the partial switching of ferroelectric domains in an FTJ can be precisely controlled, multiple intermediate resistance states can be obtained. This renders FTJs a suitable candidate for artificial neural network (ANN) synapses. For example, Guo et al. [65] successfully used interface engineering for the modulation of biological synaptic plasticity in an FTJ with BaTiO_3_ serving as a tunneling layer; they also implemented the learning rule that is typical for biological synapses, namely STDP. Other synaptic functions—such as facilitation, potentiation, and depression—have been successfully demonstrated in other FTJs [49]. This section introduces some typical research on FTJ synapses from both inorganic and organic perspectives.

**Fig. 4:**
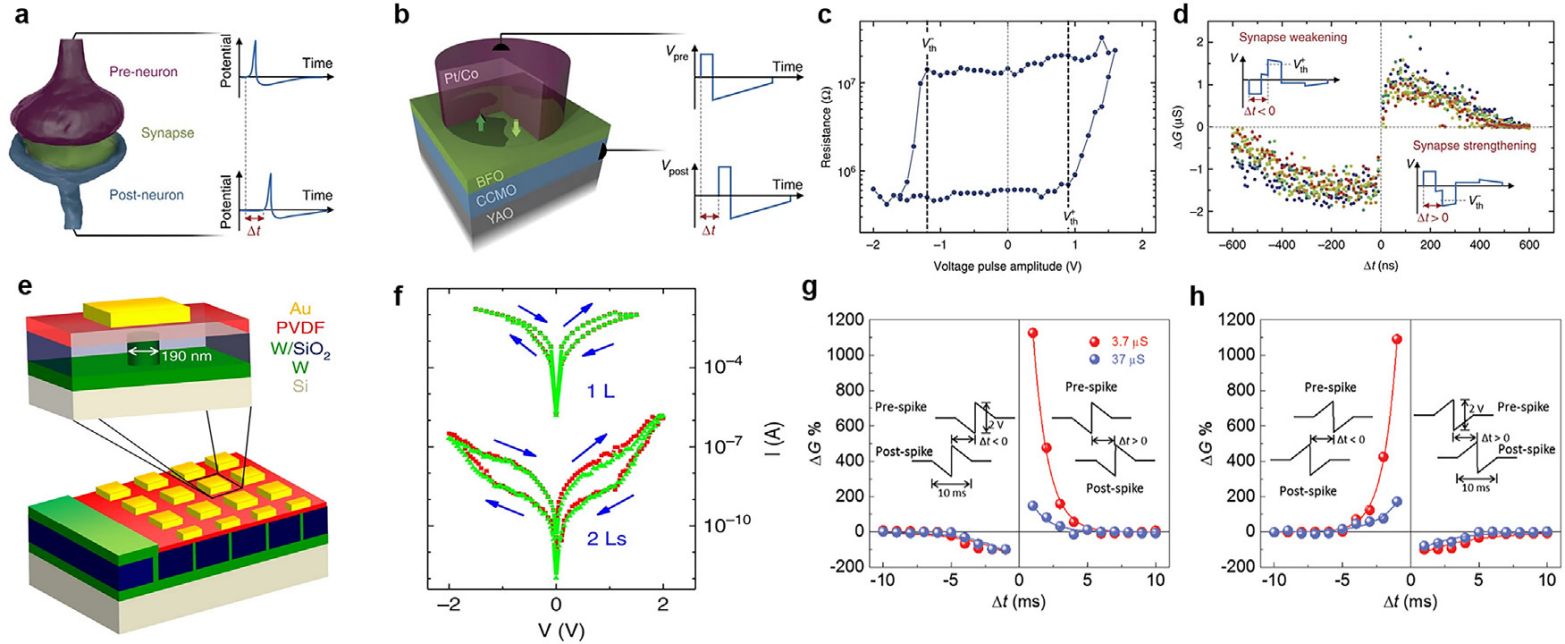
**Synaptic devices based on inorganic and organic FTJs.** (a) Schematic of a biological synapse. The synaptic transmission is modulated by the causality Δt of neuron spikes. (b) Ferroelectric memristor in which BiFeO_3_ serves as a tunneling layer. YAlO_3_ is used for the substrate, and (Ca, Ce)MnO_3_ and Pt/Co are employed for the bottom and top electrodes, respectively. (c) Hysteresis of the resistance versus voltage amplitude at a single pulse. The threshold voltages (*V*_th_) in the on and off states of the device are clearly indicated. (d) Regulation of the conductance *ΔG* at the delay time *Δt* between the pre- and post-synaptic spikes, clearly reflecting the STDP learning rule for this FTJ. The inset displays the superposition waveforms (*V*_pre_ - *V*_post_) generated when both pre- and post-neuron spikes reach the memristor with a delay Δt [66] (Copyright 2017 Springer Nature). (e, f) Schematic of a PVDF FTJ array and the *I*–*V* curves of the one- and two-layer FTJs [11] (Copyright 2016 Springer Nature). (g, h) Demonstration of asymmetric STDP based on Hebbian learning for the organic FTJ. The FTJ structure has Nb-doped SrTiO_3_ as a substrate and P(VDF-TrFE) as a tunneling layer. The insets depict the timing difference of pre- and post-spike [67] (Copyright 2019 John Wiley and Sons).

Numerous inorganic FTJs have been proposed. Boyn et al. [66] demonstrated that an FTJ with BiFeO_3_ serving as the tunneling layer has typical synaptic plasticity. They proposed a physical model of nucleation-dominated reversal of domains and successfully applied the model to conductance changes in memristors. To achieve behavior similar to that of biological synapses, they successfully simulated the spiking of pre- and post-neurons by applying a series of rectangular voltage pulses to the structure, as shown in Fig. 4a,b. Fig. 4c shows the relationship between the junction resistance and voltage pulse amplitude. Their results indicated that a device can be switched between high- and low-resistance states by simply applying an excitation that exceeds the voltage threshold *V*_th_. When the time difference between the pre- and post-neuronal spikes arriving at the memristor is the delay Δ*t*, the corresponding superposition produces the waveform (*V*_pre_–*V*_post_) (Fig. 4d). Specifically, when Δ*t* > 0, the resulting combined waveform increases the FTJ's conductance (synaptic weight enhancement); conversely, when Δ*t* < 0, it reduces the FTJ's conductance (synaptic weakening) [66]. The conductance change of FTJs can be described by a well-established nucleation-limited model owing to the presence of ferroelectric switches. On the basis of this model, Boyn et al. [66] conducted STDP simulations and obtained various distinct learning curves. Additionally, by adding the extracted parameters to the model and applying different voltage waveforms to each device, they predicted the conductance variations for the STDP types involved. Their experimental results revealed that the model predictions were consistent with the actual measured conductivity changes. They subsequently demonstrated unsupervised learning in an SNN by using this physical model as a simulation platform. In the neural network composed of a crossbar array with this FTJ, the images were encoded by the input neurons, and the output neurons were excited after the threshold was reached. After multiple training epochs, the network achieved an exceptional recognition rate. This research paves the way for in-memory computing technologies that involve low-power hardware and offer high-density integration.

Organic FTJs have considerable potential for use in silicon technology, large-area applications, and flexible electronic devices; they are also potential structures for realizing high-performance devices with non-volatile memory. Furthermore, organic FTJs exhibit distinct electron transport properties from inorganic FTJs because of their weaker van der Waals interfacial bonding. Tunneling conductance generally exhibits an exponentially decaying relationship with the ferroelectric layer thickness, thus limiting the development of FTJs at the nanoscale. However, Tian et al. [11] were the first to utilize ferroelectric PVDF films with a thickness of only a few nanometers as the barrier structure in the tunnel junction. The corresponding PVDF FTJ structure is illustrated in Fig. 4e. PVDF ferroelectric films can achieve reversible switching of polarization states at several monolayer scales and multilevel storage states (Fig. 4f). Tian et al. [11] observed that the ratio of ferroelectric polarization regulation to the tunneling current exceeded 1000%. This provides a basis for exploring tunneling electrons and the coupling-related properties of polarization. Since the study of Tian et al., additional studies on organic FTJs have been conducted. Majumdar et al. [67] used a 3-nm-thick ferroelectric polymer layer to simulate organic tunnel connections and achieve synaptic plasticity. By resetting the small tunneling current at the nanosecond timescale, they simulated the behavior of memristors with multilevel conductance states and low-energy operation. Some synaptic functions such as long- and short-term synaptic plasticity, paired-pulse facilitation, and programmable synaptic weights were also simulated. As displayed in Fig. 4g,h, both Hebbian and anti-Hebbian learning rules are achieved. The findings of the two aforementioned studies indicate promising prospects for the application of organic FTJs as biomimetic synaptic devices.

#### 2.2.4 FeFETs and FeS-FETs as synapses

**Fig. 5:**
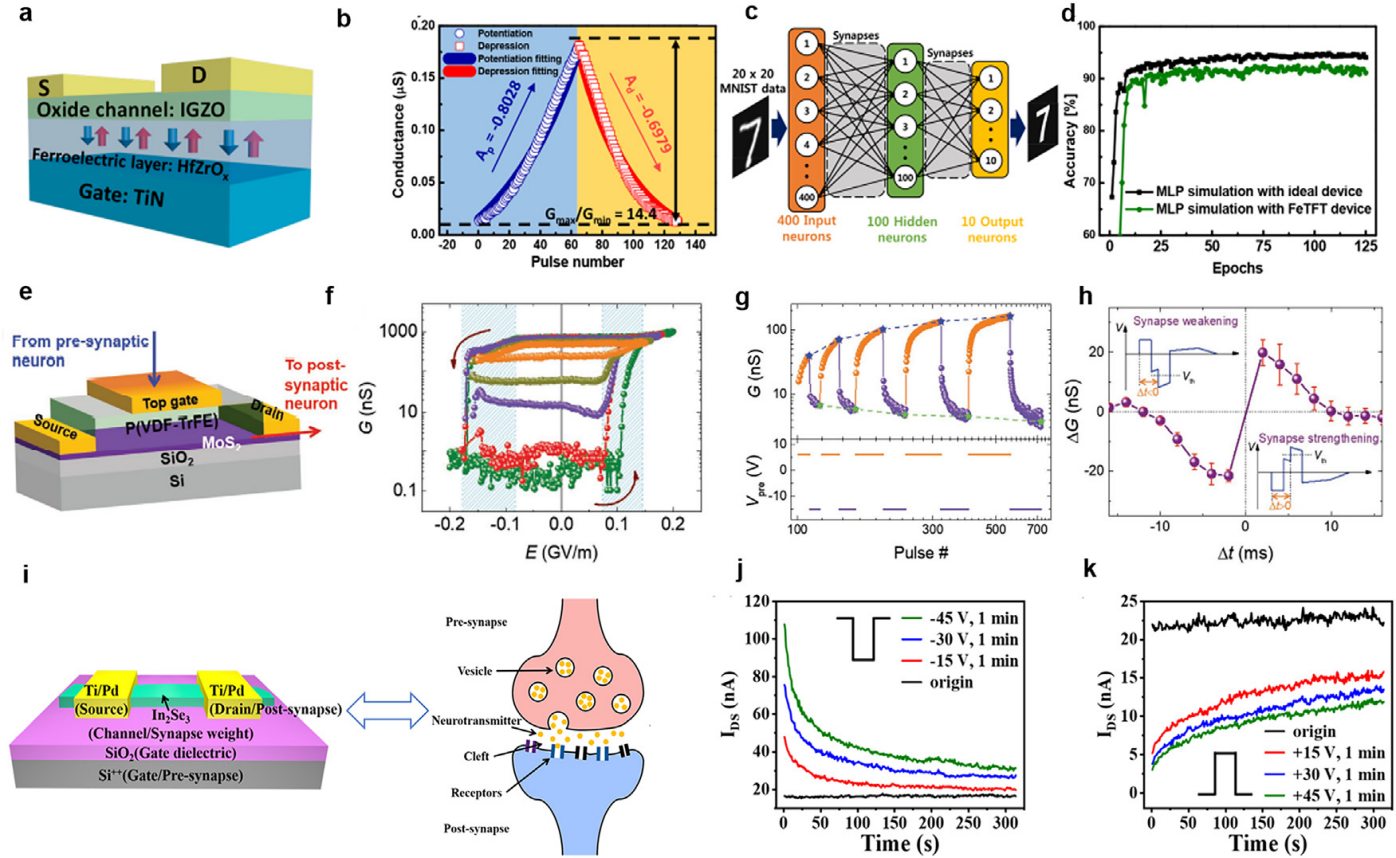
**Artificial synapses based on FeFETs and FeS-FETs.** (a) FeTFT in which HfZrO_x_ is used as the gate dielectric layer and IGZO is employed as the channel material. (b) Corresponding potentiation and depression properties of the device. (c) Schematic of a neural network containing a hidden layer. (d) Simulation results regarding the image recognition accuracy of the network composed of the FeTFT, which is comparable to that of the ideal case [68] (Copyright 2019 American Chemical Society). (e–h) Schematic of an artificial synaptic device based on an organic FeFET [P(VDF-TrFE)/MoS_2_] and (f) its corresponding *G*–*E* hysteresis curve. Typical synaptic plasticity, LTP/D (g) and STDP (h), was simulated using this transistor. The inset displays the superposition waveforms (*V*_pre_ - *V*_post_) produced when both pre- and postsynaptic neuron spikes reach the device with a delay time Δt [69] (Copyright 2018 John Wiley and Sons). (i) FeS-FET for simulating biological synapses in which the channel material is two-dimensional In_2_Se_3_. Response of the device to the application of negative (j) and positive (k) gate voltage spikes [70] (Copyright 2020 American Chemical Society).

Ferroelectric transistors are regarded as excellent candidates for data storage and biomimetic synapses because their channel conductance can be precisely modulated by the non-volatile polarization state of ferroelectric materials. Moreover, their channel conductance modulation mechanism can solve problems related to nonlinear weight updating and a small switching ratio in synaptic devices. Since the proposal of the FeFET concept in 1963 [71], the channel conductance of FeFETs has been considered a synaptic weight value [72]. In the neuronal circuit proposed by Ishiwara et al. [73], FeFETs are used to simulate memristor behavior. Yoon et al. [74] successfully prepared electrically modifiable FeFET synaptic circuits on the basis of the aforementioned circuit. These works provide a fundamental perspective regarding the application of FeFETs containing different ferroelectric materials in future artificial synapses. In the process of synaptic plasticity learning (STDP), which is the basis of the brain's learning rule, is influenced by the close temporal association between pre- and post-synaptic neuron spikes. This mechanism was illustrated in the work of Kaneko et al. [75]. They used a Pb(Zr,Ti)O_3_-based FeFET, with the help of a CMOS selector circuit, to achieve the STDP mechanism by applying positive and negative pulses to the grid. In 2011, with the discovery of hafnium-based ferroelectricity [37], numerous HfO_2_-based synaptic transistors were developed. They have the advantages of compatibility with CMOS processes, favorable miniaturization characteristics, and easy implementation of high-density integration [76]. Kim et al. [68] prepared HZO FeFETs by using an IGZO channel (Fig. 5a)*.* The channel conductance exhibits clear hysteresis in response to the gate voltage. In this device, the change in channel conductance originates from polarization modulation in the ferroelectric gate dielectric layer. Extending incremental bias pulses engenders excellent potentiation and depression characteristics in the device (Fig. 5b). By using HZO FeFETs, Kim *et al*. [68] constructed a two-layer neural network (Fig. 5c) comprising 400 input neurons used to simulate 20×20 database data and 10 output neurons corresponding to 10 numeric categories (digits 0 to 9). For simulation, they used the multilayer perceptron (MLP) algorithm in the network to update the weights. They reported that after 125 training epochs, the neural network achieved 91.1% accuracy, which was comparable to the 94.1% accuracy obtained under ideal conditions (Fig. 5d).

Inspired by the work of Kim et al., Halter et al. [77] designed an HZO-based FeFET with good linearity; in this device, WO_x_ serves as the semiconductor channel. They demonstrated the characteristics of voltage control, symmetry, and analog potentiation and depression. They reported the device to have a short programming time (40 ns) and low energy consumption (fJ). Additionally, they reported low noise in retention measurements (1%) and that the channel's thickness and geometry could be changed to engineer on/off ratios of 1% to 200% [77]. Accordingly, this FeFET has considerable potential for use in large-scale integration of synaptic arrays.

In addition to transistors based on inorganic ferroelectric materials, organic ferroelectric transistor synapses have received extensive attention. Tian et al. [69] designed a high-performance, low-energy-consumption organic FeFET with a MoS_2_ channel and a P(VDF-TrFE) copolymer gate dielectric (Fig. 5e). In this device, the memristor modulates the MoS_2_ channel's conductance through the ferroelectric polarization-switching process and accurately achieves more than 1000 intermediate conductance states (Fig. 5f). In addition to this bipolar switching characteristic achieved through ferroelectric polarization, a change in polarity can alternately adjust the device's conductivity between an increment and a decrement; this is achieved through the application of a series of the same positive and negative pulses (Fig. 5g). Tian et al. [69] conducted experiments and observed that the device can exhibit typical synaptic plasticity, such as STDP (Fig. 5h). They determined the change in channel conductance from the delay Δ*t* between the arrival of pre- and post-synaptic neuron spikes in the device. The synaptic weights increased at Δ*t* > 0 and decreased at Δ*t* < 0. Moreover, the device was noted to consume very little energy (<1 fJ) and to have a lifetime of nearly 10 years. These experimental results strongly suggest that FeFETs based on organic ferroelectric materials have potential for use in in-memory computing technologies and for realizing large-scale neural architectures.

FeS-FETs, which have higher retention performance than do FeFETs, have also been widely applied in artificial synapses. In the α-In_2_Se_3_ FeS-FET prepared by Si et al. [62], an on/off ratio of more than 10^8^ and a large memory window were obtained. The device contains a 90-nm-thick SiO_2_ film with large EOT and a 15-nm-thick HfO_2_ film with small EOT. The range of the electric field's influence on the semiconductor layer varies between SiO_2_ and HfO_2_; the range is small for SiO_2_ and large for HfO_2_. Accordingly, Tang et al. [70] simulated the spike response of an α-In_2_Se_3_ FeS-FET under various gate pulses and demonstrated the feasibility of the structure as a synaptic device. Similar to the case in other three-terminal devices, the gate insulating layer composed of SiO_2_ is used to mimic the presynaptic membrane in biological synapses, whereas the source/drain of the device is used to mimic the postsynaptic membrane (Fig. 5i). The channel conductance of α-In_2_Se_3_ can be regarded as the synaptic weight and to be regulated by the gate voltage. To simulate synaptic behavior, Tang et al. [70] applied various numbers of pulse spikes to the gate terminals to test the response performance of the FeS-FET. Fig. 5j,k illustrate the variation in *I*_DS_ with time for voltage pulses of different amplitudes. They obtained excitatory postsynaptic current (EPSC) by applying a negative gate voltage spike and obtained inhibitory postsynaptic current by applying a positive spike pulse. As the voltage spike's amplitude increased from 15 to 45 V, the initial current was considerably different from the original current after the voltage pulse was withdrawn. This demonstrates that the degree of polarization within α-In_2_Se_3_ increases.

## 3 In-memory computing based on ferroelectric materials

In the 1940s, McCulloch and Pitts [78] established a mathematical model of neural networks. Since then, a trend has emerged in machine learning, which entails simulating the transfer of information between biological neurons. ANNs are subsets of machine learning and are used to map patterns of connections in integrated circuits, which are analogous to the connections between biological neurons in the human brain [79]. An ANN generally comprises layers containing different numbers of nodes, including an input layer, one or more hidden layers, and an output layer. Each layer contains multiple neurons and connects to neurons in other layers. The informational interaction that occurs during connections can be considered the weights in biological synapses. The data transmitted between different layers depend on whether the output of a single node reaches a threshold value [79,80]. In recent decades, scholars have been inspired by biological neural systems and have extensively developed neural networks that have been widely used in many artificial intelligence (AI) applications, including autopilot systems, image analysis, robotics, and speech recognition. However, compared with those in real biological neural networks, the neurons and synapses in current ANN models are greatly simplified in terms of applications. Various network structures have been developed on the basis of different connection structures. In addition to the earliest perceptron networks, the most commonly used neural networks currently include: (1) deep neural networks (DNNs) and convolutional neural networks (CNNs), which are feedforward neural networks; (2) recurrent neural networks (RNNs), which are feedback neural networks; and (3) SNNs, which represent the new generation of neural networks [81]. This section presents a summary of research on ferroelectric synapses used in memory devices. The section discusses the existing ferroelectric in-memory computing technology as classified by these four types of ANN.

### 3.1 Ferroelectric DNNs

**Fig. 6:**
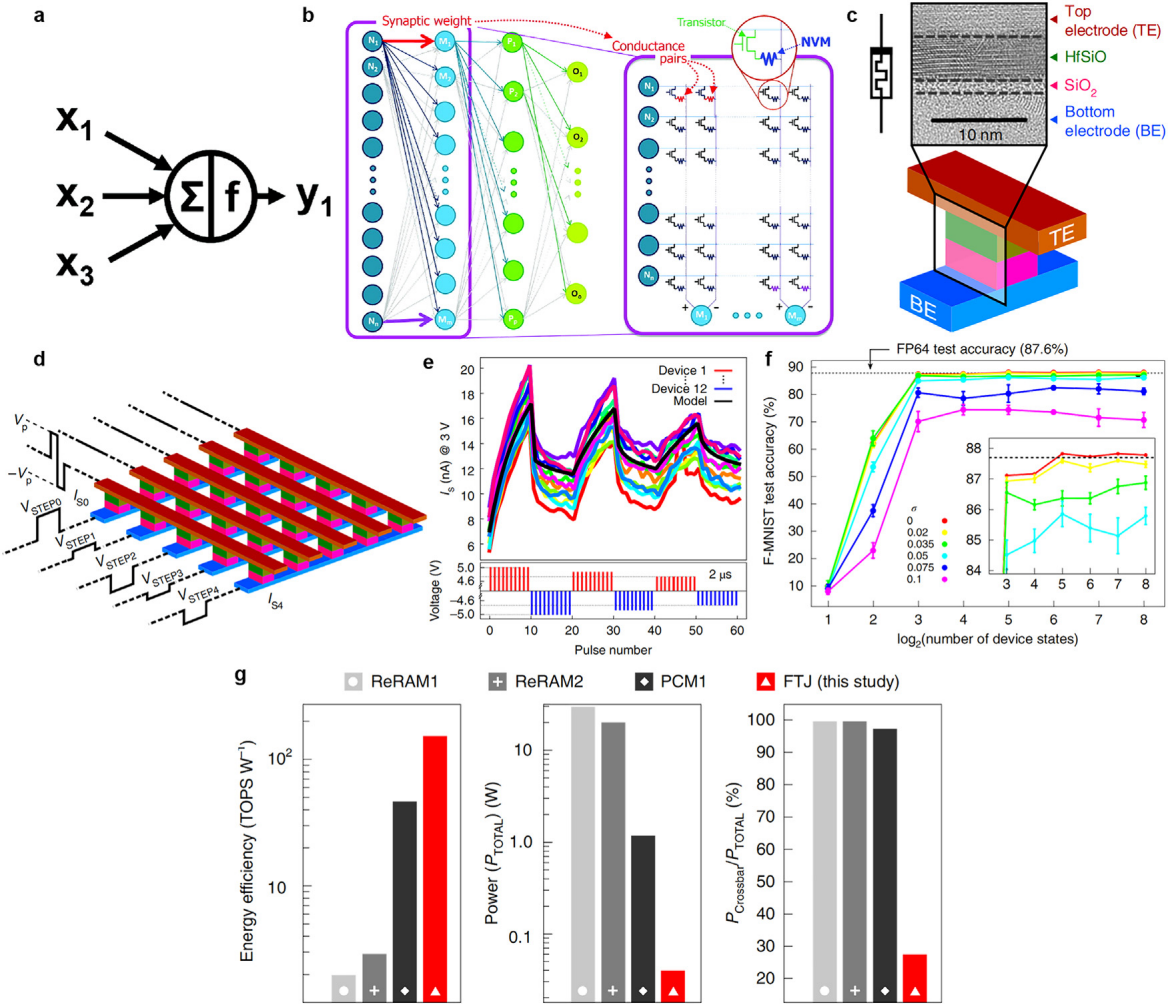
**Schematic of DNN and the application on ferroelectric memory devices.** (a) Firing of downstream neurons depends on the weights of the firing of all upstream neurons, and the evaluation is executed in multiply–accumulate (MAC) mode. (b) Forward inference and backpropagation of the DNN combined with MAC operation can achieve matrix–vector multiplication on large non-volatile memory arrays [83] (Copyright 2016 Informa UK Limited). (c) Sketch of the FTJ device structure based on a Si-doped HfO_x_. (d) The 5 × 5 nonselective (passive) FTJ crossbar array (where *V_p_* is the amplitude of the word line biphase write pulse). (e) Relationship between *I*_g_ of the 12 devices and the number of pulses at different writing pulse amplitudes, showing distinguishable bipolar switching. (f) Inaccurate weights of the neural network computed using FP64 are transferred to the FTJ crossbar array, the inset depicts the test accuracy of the local amplification. (g) Comparison of computing efficiency between the FTJ device and other memory devices [84] (Copyright 2020 Springer Nature).

A DNN comprises neurons, which are parallel processing units connected by plastic synapses. The essential idea is to stack multiple layers of neurons; each layer extracts specific features and information, and the output information from this layer is used as an input signal that is transmitted to the next layer [82]. Thus, the input information can be hierarchically expressed. The firing or spiking of downstream neurons depends on the weights of the firing of all upstream neurons (Fig. 6a) [83]. DNNs are usually trained using a gradient-descent-based supervised learning algorithm. During training, the input data are transmitted between layers of neurons and propagated forward, and matrix–vector multiplication operations are executed in the synaptic network. Ohm's law is used to perform multiplication at each intersection, and Kirchhoff's current law is employed to obtain the summation of currents along a row or column (Fig. 6b) [83]. The final layer's response is compared with the input data labels; thus, the error between the two can be backpropagated, and the subsequent error can be reduced by updating the synaptic weights.

Berdan et al. [84] performed linear calculations of ultralow currents through nonlinear FTJ memristors, and they demonstrated the feasibility of performing analog-voltage-amplitude vector–matrix multiplications in a selector-less FTJ crossbar array. As displayed in Fig. 6c, the FTJ device has an ultrathin (∼4 nm) Si-doped HfO_x_ layer and a thin SiO_2_ layer between the upper and lower electrodes. The device was manufactured in accordance with the standard CMOS process. Moreover, the device can achieve analog conductance modulation with the altering of pulse amplitude (Fig. 6e). On the basis of this single device, they fabricated a 5 × 5 passive FTJ crossbar to implement a device-aware pulsing algorithm, which can program passive FTJ crossbars in parallel and line by line (Fig. 6d) [84]. They constructed a multilayer DNN, trained it using the Fashion-Modified National Institute of Standards and Technology (MNIST) task, and mapped the training weight to the FTJ conductance range. The weight of the full-precision training network was divided into multiple conductance levels on the basis of increasing variation σ (Fig. 6f). They found the accuracy in the full-precision test to be 87.6% with only 5 bits of weight precision. The energy consumption of this network was reported to be much lower than that stated in other papers (Fig. 6g).

### 3.2 Ferroelectric CNNs

**Fig. 7:**
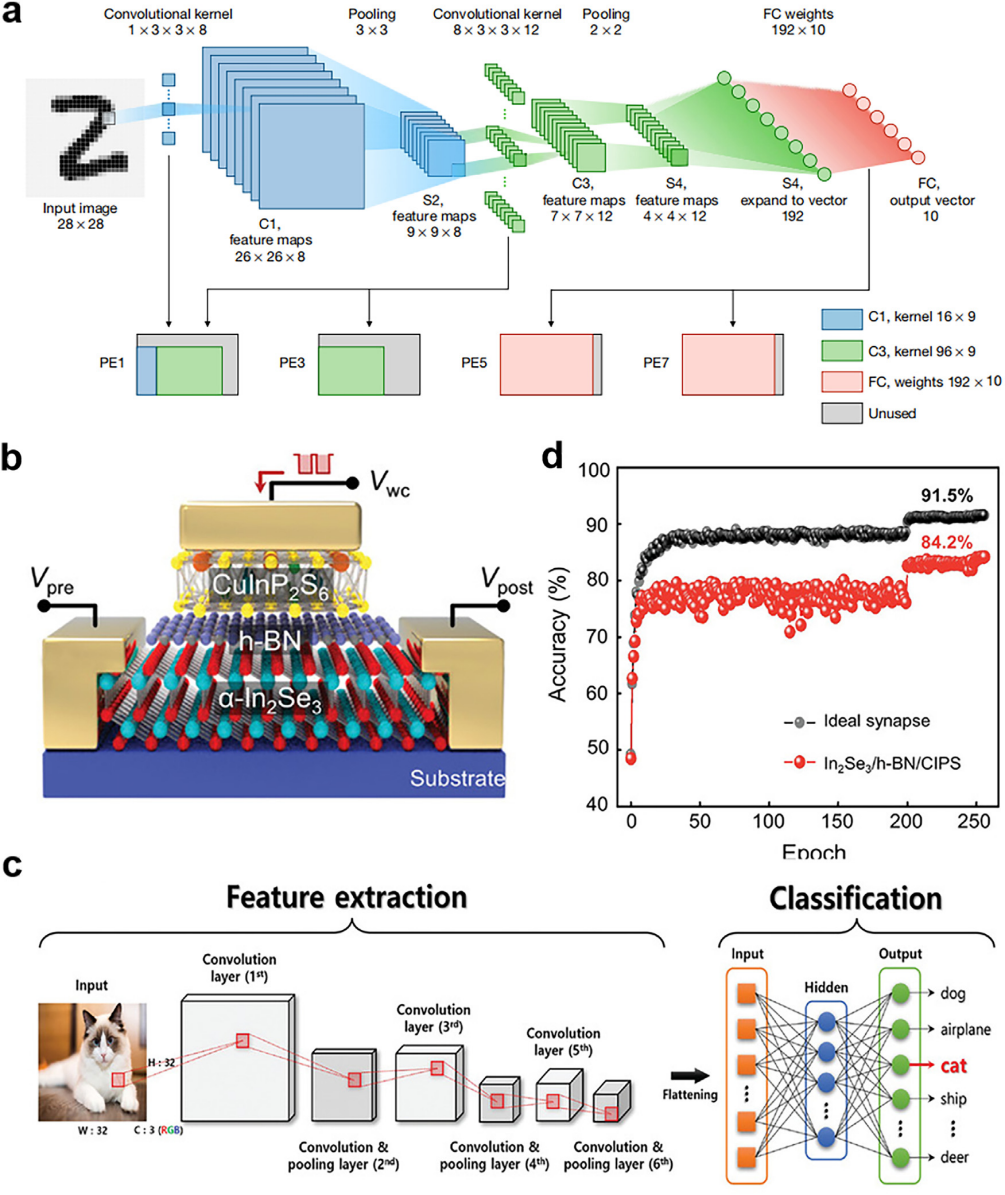
**Schematic of CNN and the application on ferroelectric synaptic devices.** (a) Five-layer CNN structure for MNIST image recognition, including convolution, pooling, nonlinear, and fully connected layers [87] (Copyright 2020 Springer Nature). (b) Structure of FE-FET synaptic device integrated with the vdWH CuInP_2_S_6_/α-In_2_Se_3_. (c) Schematic of a CNN used for image recognition and classification. (d) Recognition accuracy versus number of epochs. The structure has high accuracy comparable to that of an ideal synaptic device [93] (Copyright 2022 John Wiley and Sons).

Inspired by the visual system of the human brain, which recognizes the world by perceiving local to global information, scholars developed CNNs, a type of feedforward neural network with major advantages in many applications, including image retrieval, target localization detection, and speech recognition [85]. Fig. 7a presents the structure and information processing of a CNN. First, the convolutional layer extracts the features in the input information map and uses the convolution kernel to obtain the product. By summing the operations of the corresponding elements, the network maps the receptive field information to the elements in eigenmaps. Throughout the process, pooling layers are continuously used to reduce the dimension of the input graph and compress the amount of data. Nonlinear features are then introduced into the network. Finally, the results are identified and classified by the fully connected layer of the network [86,87].

The conventional convolution operation in a CNN is performed by computational and graphical processing units, which consume considerable power, limiting the application of CNNs in consumer electronic products [87]. Current CNNs are implemented using cross-point arrays composed of novel memory storage devices such as phase change memory (PCM) [88,89], resistive RAM [90] and ferroelectric memory device [91,92], which speed up the convolution operations and improve the accuracy and efficiency of digital implementation. Baek et al. [93] simulated biological synaptic dynamics by using FeFET synaptic devices integrated with van der Waals heterostructures (vdWHs) composed of CuInP_2_S_6_ and α-In_2_Se_3_ (Fig. 7b). They demonstrated a CNN comprising six convolutional layers for feature extraction and two fully connected layers for classification (Fig. 7c). They defined the synaptic weight as the difference in conductance between two equivalent ferroelectric synapses, and they observed that the model achieved an image recognition accuracy rate of 84.2% (Fig. 7d) after training and inference. These results demonstrate the extensive potential of vdWH FeFETs as a pioneer of 2D ferroelectric devices in the AI era as well as their potential for use in in-sensor computing technology.

### 3.3 Ferroelectric RNNs

**Fig. 8:**
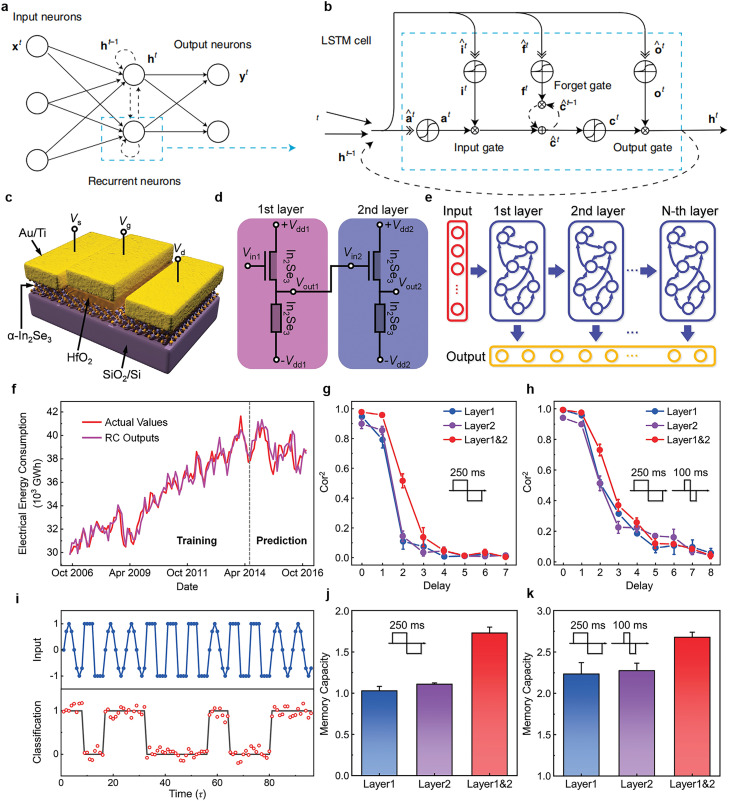
**RNN structure diagram and demonstration of multilayer RC based on α-In_2_Se_3_ FeS-FET for hierarchical information processing.** (a) Sketch of an RNN in which the anterior and posterior inputs are correlated. (b) LSTM, a specific form of RNN; the weight of the self-loop is increased by adding various gates, such as input, forget, and output gates [94] (Copyright 2019 Springer Nature). (c) Sketch of an FeS-FET structure based on α-In_2_Se_3_, in which α-In_2_Se_3_ serves as both the channel and gate insulator. (d) Circuit schematic of a two-layer RC system. (e) Deep RC architecture consisting of multiple standard RC units stacked in sequence. (f) The time-dependent sequence prediction tasks of the electrical energy consumption in Brazil performed by the designed RC architecture are presented. (g, h) Relationship between Cor^2^ and the delay for a pulse width of (g) 250 ms and (h) 100 and 250 ms. Cor^2^ was used to quantify the MC. (i) Waveform classification tasks (sine wave and square wave) performed by the multilayer RC system. (j, k) MC calculated from the results of (g) and (h), respectively [98] (Copyright 2022 John Wiley and Sons).

Unlike feedforward neural networks, which pass information directly forward (without recontacting nodes that have already been passed), an RNN uses not only the current input information but also the previous input information [81]. Neurons are interconnected or self-connected, and information can flow both into the network and cyclically (Fig. 8a). Thus, the previous information (at time step *t* *−* 1) can affect the decisions at time step *t*. This consequently enables the processing of sequential data that vary over time. However, because the layers and time steps of a neural network are interrelated through multiplication, exploding exponential weights or vanishing gradients can easily arise. Standard RNNs cannot solve such problems and are thus insensitive to long-term temporal correlations. Researchers have proposed a new type of RNN, namely long short-term memory (LSTM) networks, to prevent the problem of gradient disappearance (Fig. 8b) [81,94]. LSTM networks store information in gated cells without the information passing through the normal information flow; this helps to preserve errors. However, LSTM-based RNNs constructed using this digital strategy have a complicated structure and are limited by the communication bandwidth and computational efficiency [94].

Apart from LSTM-based RNNs, researchers have proposed a simplified RNN form called reservoir computing (RC), which was developed from the echo state network [95] and liquid state machine [96]. An RC network consists of three layers of neurons: the input and output layers and a middle layer, namely the reservoir layer [97]. The reservoir layer is the most complex and crucial part of the whole RNN, and its internal neurons are sparse, random, and fixed. The advantage of RC is that the weights of the reservoir itself do not have to be directly trained; their settings are based on a few parameters that control the properties of the connection graph. The problem of vanishing or exploding gradients inherent in backpropagation algorithms can be bypassed in RC [1].

Recently, Liu et al. [98] constructed a stackable reservoir system by using ferroelectric α-In_2_Se_3_ FeS-FET devices. Fig. 8c,d show the system's basic structure (constituting stackable RC) and a circuit schematic of the multilayer RC system, respectively. The system establishes a connection between the reservoir level and the multilayer RC network through resistance matching and voltage distribution between the FeS-FET and the flat device manufactured on the same α-In_2_Se_3_ sheet. In the deep RC architecture (Fig. 8e), the first layer receives the input signals and sends its output signal to the second layer for processing and also to the output layer. This process is then repeated; thus, the input information is continually processed. Liu et al. [98] demonstrated their system's performance in the prediction of time-dependent sequences (Fig. 8f). The results revealed that the multilayer RC system could predict the electric energy consumption of a country such as Brazil, indicating that it has the ability to map the input to a high-dimensional space. They further demonstrated their system's performance in waveform classification (Fig. 8i).

The system developed by Liu et al. [98] includes a hierarchical structure comprising two layers for performing the prediction and classification tasks; therefore, the system can represent features on a multitemporal scale. The memory performance of the system was evaluated using memory capacity (MC), which can be derived by summing the squared coefficients of the correlation *Cor^2^* between different delays. When the two layers were used, the *Cor^2^* value decreased as the delay increased; similar results were obtained when only the first or second layer was used (Fig. 8g). The MC for the multilayer output was considerably superior to that for the single-layer output owing to the multitemporal scale characteristics (Fig. 8j). Fig. 8h,k show that in all the three cases, the MC can be improved by adding one more modulation pulse width, with the *Cor^2^* values having the same trend. The work of Liu et al. [98] is promising for the development of deep RC architectures and for the physical implementation of hierarchical-information-processing systems.

### 3.4 Ferroelectric SNNs

**Fig. 9:**
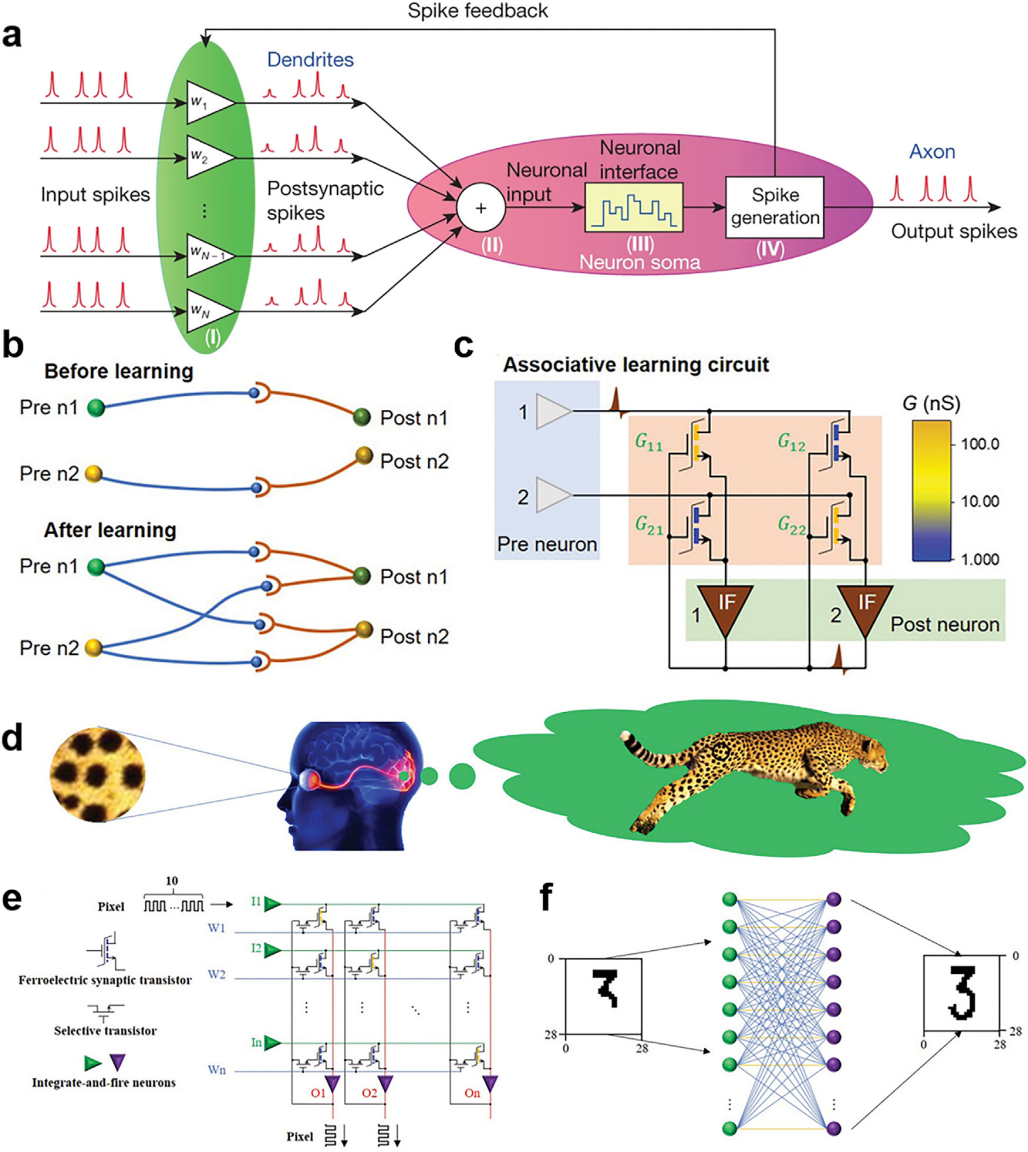
**Schematic of SNN and the use for associative learning based on ferroelectric synaptic transistors.** (a) Schematic of an SNN, which generates neural output spikes only when the integrated power of the postsynaptic spikes exceeds the threshold [99] (Copyright 2019 Springer Nature). (b) In associative learning, neurons can make new connections with multiple next-level neurons. (c) Diagram of an electronic circuit inspired by associative learning in which the main component units are ferroelectric transistors and integrate-and-fire neurons. (d) Schematic of human associative memory; a pattern is associated with a leopard's distinctive spots. (e) Neural network circuit architecture for associative learning. (f) Two-layer neural network for handwritten digit recognition [101] (Copyright 2021 John Wiley and Sons).

SNNs are the most evolved neural network model after CNNs; they were developed by incorporating the concept of time into neural networks, in addition to the neurons and synapses present in conventional neural networks. SNNs are typically propagated using discrete events, called spikes, as opposed to the normal consecutive forms of values. SNN nodes do not fire in periods, as a perceptron does, but must wait for the membrane potential to reach a threshold [1,81]. The neurons in an SNN are strongly interrelated, and when triggered, an individual node passes on the signal to other nodes and uses it to regulate the decay of its own potential [99]. The signal in an SNN consists of a sequence of discrete spikes (Fig. 9a). The time, amplitude, and frequency of the spike signals are the carriers of information and are fed back by spikes through Hebbian STDP [99]. Therefore, compared with the first two generations of neural networks (perceptron and backpropagation neural networks), SNNs exhibit higher time and energy efficiency.

Associative learning, as a type of SNNs, is a pervasive learning mechanism in biology that reflects individuals’ close relationship with their surroundings [100]. If different individuals share some common features, an intrinsic connection between them can be discovered. Such connections can vary according to numerous factors, such as the duration of the applied stimulus and the frequency of application. Three-terminal devices are particularly suitable as a hardware base for associative learning owing to the feedback regulation of the channel's conductance (synaptic weights) through the gate terminal [16,48]. Yan et al. [101] proposed a ferroelectric synaptic transistor with 2D MoS_2_ as the channel and the copolymer P(VDF-TrFE) as the gate dielectric. The transistor achieves associative learning by using an SNN and integrate-and-fire neurons. In this transistor, short-to-long-term memory conversion and STDP/SRDP can be realized on a single synaptic transistor; moreover, adaptive coupling between neurons can be achieved using weighted control terminals to modulate ferroelectric domain dynamics. Yan et al. [101] successfully demonstrated the conditional reflex experiment of Pavlov. Fig. 9b,c illustrate the proposed associative learning rule and the circuit designed for associative learning. Associative learning reflects a conditional reflex and can be used to associate the whole information from part of the information. Fig. 9d shows a pattern within a circle; upon seeing this pattern, a person will associate it with the unique spots of a leopard. Integrating this function of the human brain into neural networks can considerably improve the recognition ability of AI systems in harsh environments, such as those involving face recognition for people wearing a mask and those involving license plate recognition for stained license plates. Yan et al. [101] also used the aforementioned neural network to perform associative learning for multiple numbers. Fig. 9e,f show the actual circuit structure based on this synaptic device and the corresponding two-layer neural network for handwritten digit recognition, respectively. The voltage pulse sequence represents the pixels in the digital image. They observed that the neural network can output a complete image of the corresponding number even if the input is an incomplete image [48]. This study demonstrates the strong performance of ferroelectric synaptic transistors—through their self-learning function—in real-time information processing in complex environments, thus laying a foundation for the future development of in-sensor computing technology.

## 4 In-sensor computing technology based on ferroelectric materials

In-memory computing architectures overcome the limitations engendered by the memory wall problem that is inherent in the von Neumann computing architecture [102]. Inspired by the human brain, in-sensor computing architectures, which integrate self-adaptive sensors or multiple sensors with memory and computing functions, are also being explored. Compared with conventional architectures, all-in-one sensor computing consumes less power; results in lower latency during data transfer; and integrates the perception, storage, and computation of various simulation signals (i.e., optical, pressure, gas, sound, and heat signals) [103]. In the context of AI, cloud computing, and the Internet of Things, the amount of data generated each day is growing exponentially. Developing and exploring technologies for in-sensor computing are thus essential. Solid-state devices for in-sensor computing can be roughly classified into phase types (PCM synapses, FeFETs, magnetoresistive RAM, and optical synapses) and ion types (ion-type synapse transistors and memristors) [5,9,81]. Neuromorphic electronic devices regulated by ionic liquids and ionic gels are widely used to simulate biological synapses because their regulation principle is similar to the ion regulation mechanism in biological synapses [104,105]. However, the ionic mechanism cannot achieve sufficient or stable nonvolatility, making it unsuitable for long-term storage. By contrast, ferroelectric materials are adequately non-volatile and exert multifield modulation effects, making them promising candidates for the integration of arrays in various types of in-sensor computing [5,18].

### 4.1 Optical in-sensor computing technology

**Fig. 10:**
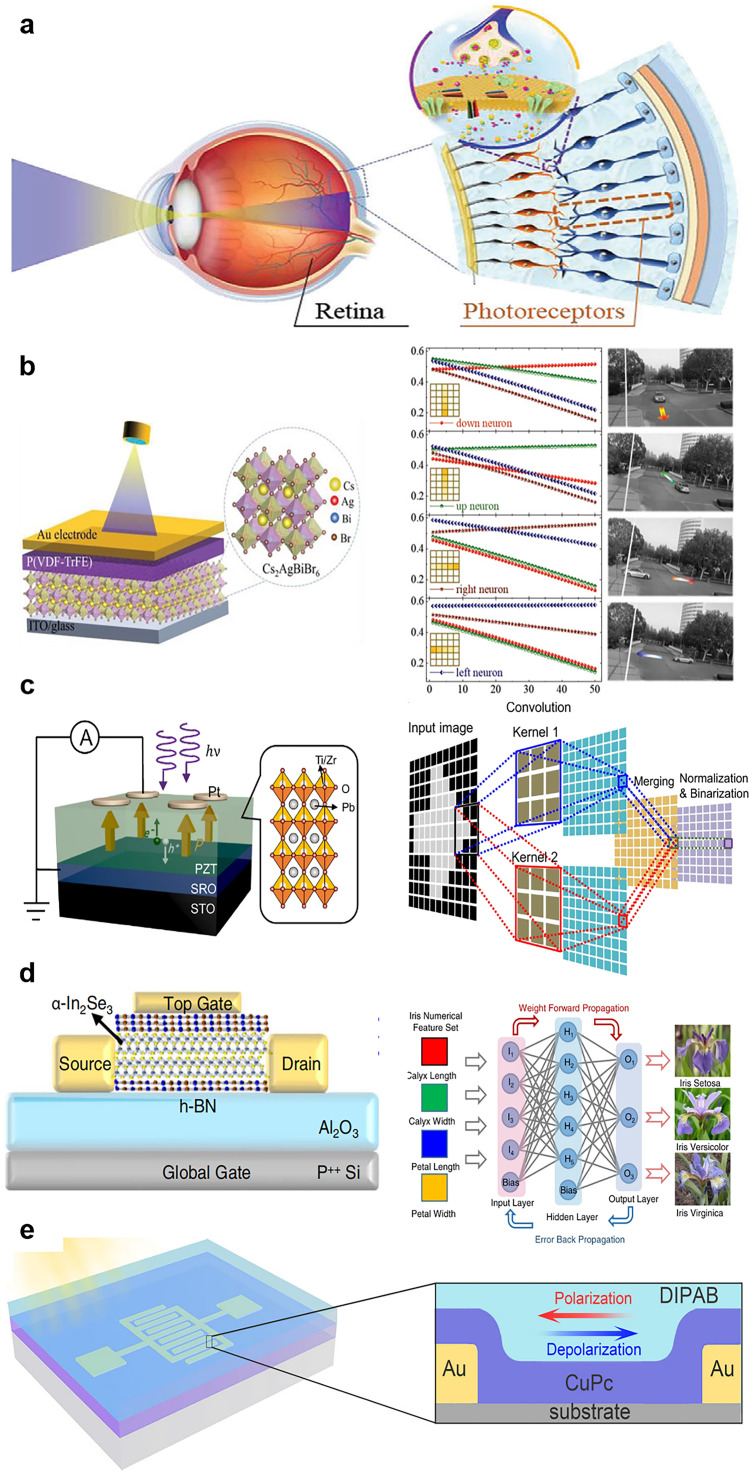
**Bio-inspired visual in-sensor computing devices based on ferroelectrics.** (a) Visual system consisting of the retina and photoreceptors. (b) Implementation of a P(VDF-TrFE)/Cs_2_AgBiBr_6_-based photonic synapse (left panel). An array containing this synapse could be used to detect the traffic flow at intersections (right panel) [106] (Copyright 2022 John Wiley and Sons). (c) Device structure of an FE-PS and the operations for edge detection [109] (Copyright 2022 Springer Nature). (d) Schematic of ferroelectric channel transistors based on 2D α-In_2_Se_3_ (left) and the neural networks for iris recognition and classification (right) [64] (Copyright 2021 Springer Nature). (e) Schematic of the structure of a two-terminal optoelectronic memristor based on molecular ferroelectric DIPAB and CuPc thin films [110] (Copyright 2022 Springer Nature).

As shown in Fig. 10a, it illustrates the human retina, in which photoreceptor cells receive and convert optical signals into electrical signals that are then transmitted to other retinal cells (bipolar, amacrine, and ganglion cells) [106]. The information is then further processed by the optic nerve. Visual signals are ultimately processed by the visual cortex, and the results are sent to other areas of the brain. By contrast, image perception, processing, and storage are separated in traditional computer-based vision systems; hence, such systems generate redundant data, which considerably reduces the speed at which perceptions can be made and also increases power consumption. Recently, researchers have proposed a new neuromorphic vision system that has low latency and high energy efficiency, similar to those of human vision; the system performs in-sensor computing and has integrated optoelectronic synaptic devices. In-sensor visual systems based on ferroelectric materials—such as HfO_2_-based oxides, the 2D ferroelectric semiconductor CuInP_2_S_6_ or In_2_Se_3_, and the traditional perovskite PZT or organic ferroelectric PVDF—are being extensively investigated [10,[106], [107], [108], [109]]. Although bioinspired visual systems have been extensively researched, developing optical in-sensor computing technology for practical applications is still warranted. The efficiency and accuracy of image recognition and motion detection must be improved, and the energy consumed during image processing must be reduced. Furthermore, the light response of an integrated sensor over a wide range of scenarios, such as scenarios involving both infrared and visible light and those involving dark environments, could be improved. Finally, the external circuitry for large-scale integration and the overall system must also be developed.

#### 4.1.1 In-sensor computing based on organic and inorganic ferroelectric materials

Lao et al. [106] proposed an in-sensor RC system based on a self-powered photovoltaic device composed of the ferroelectric polymer P(VDF-TrFE) and the inorganic lead-free double perovskite Cs_2_AgBiBr_6_. The device can simulate photoreceptor cells and synaptic functions in the human visual system (Fig. 10b). The P(VDF-TrFE) layer acts as a modulator of optoelectronic responses. An energy potential well is created at the interface between the Cs_2_AgBiBr_6_ and ferroelectric layers by inserting the Cs_2_AgBiBr_6_. This potential well makes it more difficult for photogenerated carriers to migrate to the gold electrode, which substantially extends the lifetime of the EPSC after the light stimulus has been removed. Additionally, it provides efficient nonlinear coupling for stimulating optical signals. The EPSC amplitude and its coupling strength can be tuned by adjusting the shape of the energy potential well and changing the ferroelectric polarization direction. This coupling effect is necessary for the self-powered photonic synaptic device to form in-sensor RC. The sensor reservoir can be applied to static and dynamic vision tasks. Its recognition rate when applied to static face image classification was discovered to be as high as 99.97%, and the accuracy of recognition of dynamic vehicle flow was 100%. A reserve pooling layer comprising a 5 × 5 self-powered photonic synaptic device array was used to map and collect information on the motion of vehicles at an intersection (Fig. 10b) [106]. According to the findings of this study, applying self-powered optoelectronic devices to in-sensor RC can considerably reduce the energy consumed during visual information processing and can provide a means of achieving new and effective brain-like machine vision.

Cui et al. [109] reported a new ferroelectric photosensor (FE-PS) computing network in which the strong polarization and ferroelectric photovoltaic effects of Pb(Zr_0.2_Ti_0.8_)O_3_ epitaxial thin films are exploited (Fig. 10c). The photo-response is switched through remanent polarization rather than through the gate voltage, which can further reduce energy consumption. Additionally, owing to the ferroelectric's reversible polarization, the FE-PS has a symmetrically switchable photoresponse that can be used to represent positive and negative weights. The network was discovered to achieve real-time machine vision with up to 100% accuracy. Compared with other brain-like vision hardware implementations, the network uses non-volatile polarization to regulate the photoresponse (weight) without using an external voltage, thus reducing energy consumption and obviating the need for additional memory units for storing the weight. This research opens up a wide range of applications of ferroelectric photovoltaics in machine vision hardware used to process information in real time.

#### 4.1.2 In-sensor computing based on 2D ferroelectric and molecular ferroelectric materials

In 2020, Mennel et al. [111] reported for the first time an image sensor based on a WSe_2_ 2D semiconductor photodiode array that can achieve ultrafast optical image recognition and encoding. The key feature of this bioinspired visual device is that the synapse weight varies depending on the incident light signal. By changing the photoresponse of the photodiode, the array performs optical sensing and computation. Inspired by this work, several scholars have developed devices for real-time machine vision and in-sensor computing. Wang et al. [64] proposed integrated memory and computing fusion systems that incorporate FeS-FETs with a 2D ferroelectric α-In_2_Se_3_ channel (Fig. 10d). The thermally modulated neural network shown in Fig. 10d was discovered to have 94.74% accuracy in iris classification and identification. α-In_2_Se_3_ is advantageous for large-scale integration, and it can be used to design vertically integrated circuits and achieve 3D integration through circuit stacking [112]. This can largely solve the problem of interdevice variation.

In addition to 2D ferroelectric materials, artificial synaptic and in-sensor memory devices based on molecular ferroelectric (MF) materials have received considerable attention. Cai et al. [110] proposed a diisopropylammonium bromide (DIPAB)/semi-conductor copper phthalocyanine (CuPc) interfacial memristor device based on controllable carrier injection (Fig. 10e). They were the first time to combine an MF material with a neural synaptic device exhibiting stable and controllable conductive channels. This provides a new mechanism for achieving ferroelectric polarization in hardware implementations of neuromorphic sensing and computing. Because of the excellent properties and field enhancement effect of MF materials, the interface resistance can be regulated through polarization, thus enabling the achievement of typical synaptic characteristics such as STDP. The introduction of semiconductors also confers devices with the properties of optoelectronic synapses, which can lead to high image recognition accuracy rates in sensors; this thus provides a basis for implementing an artificial vision system within in-sensor computing. Cai et al. [110] also established an optoelectronic ANN model based on MF/CuPc devices, and this model could perceive, convert, and process optical images with high recognition accuracy.

### 4.2 Tactile in-sensor computing technology

**Fig. 11:**
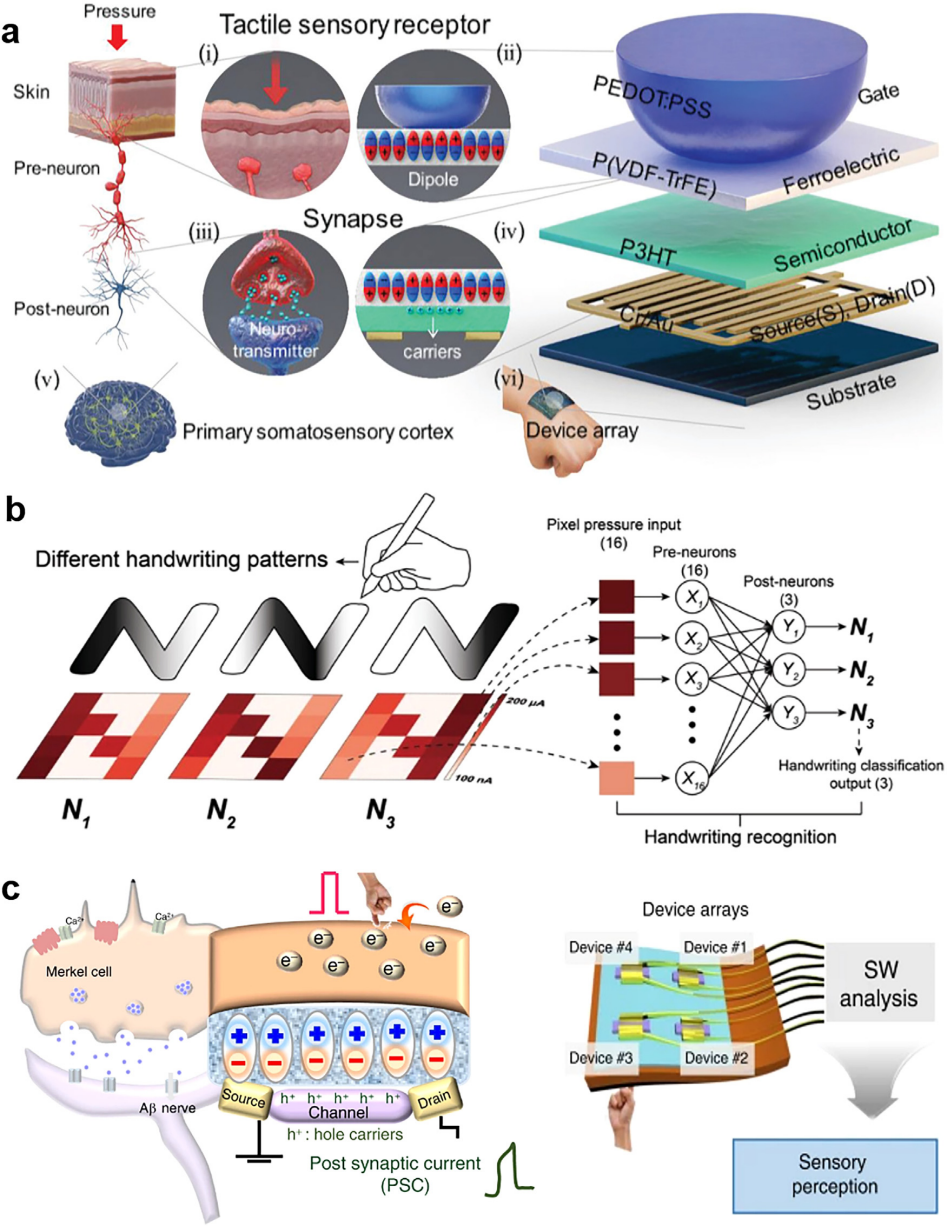
**Bioinspired tactile in-sensor computing devices based on ferroelectrics**. (a) Artificial tactile learning ferroelectric skin (ATFES) inspired by the tactile sensing system of human skin. (b) Neural network applications based on this ATFES array. From left to right: the N patterns (N_1_, N_2,_ and N_3_) of three different writing styles with corresponding networks for handwriting classification and recognition [113] (Copyright 2020 John Wiley and Sons). (c) Schematic of the sensing system corresponding to a Merkel cell and the device diagram for reception and preprocessing [114] (Copyright 2020 Springer Nature).

The tactile receptors under human skin are stimulated by external pressure and then generate response signals that are transmitted to the primary somatosensory cortex neural network; this process is the sense of touch and enables better interaction with the external environment. Inspired by this concept, scholars have developed AI electronic skin systems that can respond to various tactile stimuli. Recently, Lee et al. [113] developed a unified and tactile learning electronic skin system by using P(VDF-TrFE) FeFET arrays. The system includes a dome-shaped tactile top gate that simultaneously senses, memorizes, and learns stimuli information (Fig. 11a). The system achieved 99.66% accuracy in a classification task involving the letter N written in three handwriting styles (Fig. 11b); this demonstrates that the tactile electric skin has great potential for use in security-coding and personal identification systems. In addition, inspired by Merkel cell–neurite complexes (Fig. 11c), Lee et al. [114] developed a sensory memory system by using a nanocomposite of BaTiO_3_ and P(VDF-TrFE). They used triboelectric–capacitive coupling in ferroelectric synaptic transistors to confer the sensor with synaptic functions. The timing of touch can be autonomously predicted by analyzing the output of postsynaptic currents without connection to a neuron processor. This system's synaptic weight modulation enables reception, slow adaptation, and sensor memory. Accordingly, this system provides a new paradigm for research on pressure-based in-sensor memory.

### 4.3 Multisense in-sensor computing technology

The aforementioned artificial perception systems make decisions on the basis of a single sensory input, and this usually induces unavoidable uncertainty. Inspired by human beings’ coordination of multiple modes of perception, scholars have begun the development of artificial perception systems that can synergistically couple multiple single-mode signals to achieve more advanced and intelligent cognitive functions. If robots are to be ultra-intelligent, artificial perception systems must have advanced cognitive perception and multimodal environmental-information-processing capabilities. Researchers are striving to develop a multifunctional in-sensor computing system that can process multisensory coupled signals. Several integrated neuromorphic sensors are available, such as the visual–tactile system and tactile–olfactory system. Such integrations would improve the recognition accuracy of and reduce the data or energy consumption of sensory devices.

**Fig. 12:**
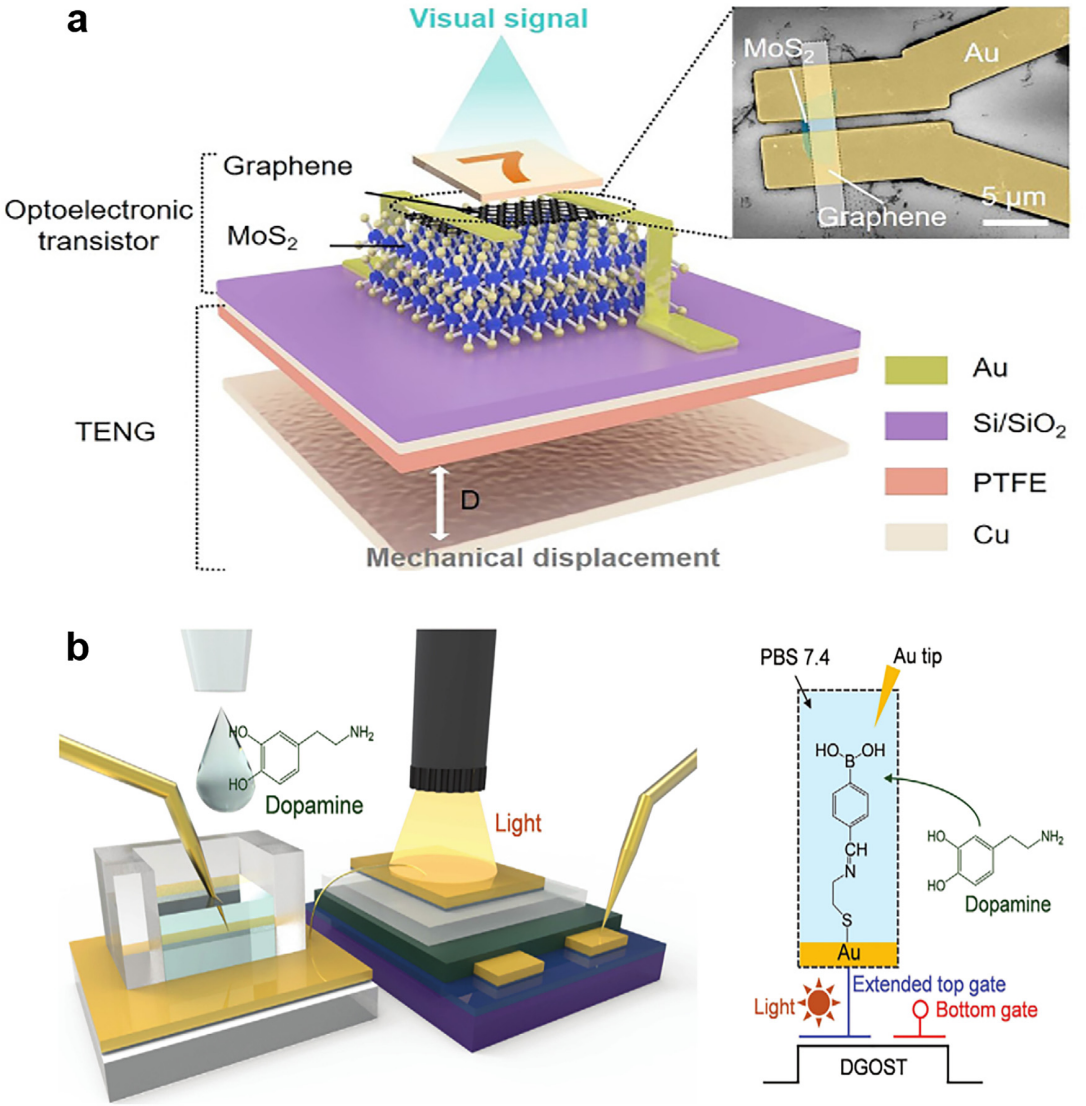
**Multisensory computing devices based on ferroelectrics.** (a) Multifunctional artificial synapse that combines mechanics with optics. The structure consists of a nanogenerator and optical synapses on the basis of graphene and MoS_2_[115] (Copyright 2021 American Association for the Advancement of Science). (b) Structural sketch of a device integrating a dual-gate organic synaptic transistor and dopamine-responsive extended-gate electrode (left) and equivalent circuit for detecting dopamine and light (right) [116] (Copyright 2021 John Wiley and Sons).

Recently, Yu et al. [115] reported a multisensory device exhibiting a graphene/MoS_2_ heterostructure and comprising an optoelectronic transistor and a triboelectric nanogenerator. Fig. 12a illustrates the structure of the dual sensory synapse. On the basis of the triboelectric potential, the charge transfer in the heterostructure can be tuned to mimic synaptic behavior. This device exhibits high image recognition accuracy (92%), which was close to the accuracy of a complex biological nervous system. Furthermore, inspired by hippocampal synapses, Lee et al. [116] designed a dual-gate ferroelectric synaptic transistor for the simultaneous detection of light and the neurotransmitter dopamine (Fig. 12b). In the hippocampal synaptic mimetic system, the remnant polarization of P(VDF-TrFE) is modulated by exposure to a dopamine solution and polychromatic light. This hippocampal synapse simulation system, which combines a chemical sensor with a light sensor, provides an opportunity to construct multisensory in-sensor computing technology. The multisensory system can effectively improve the accuracy of image pattern recognition, and it provides an avenue for the exploration of robotic sensing and perception.

## 5 CMOS-integrated ferroelectric memory for in-memory and in-sensor computing

This review reveals that ferroelectric memory devices have become a mainstream technology in neuromorphic computing. Integrating such devices into CMOS systems is imperative. Hafnium-based ferroelectric materials have attracted widespread attention because of their compatibility with CMOS processes and their potential for integration into high-density arrays [10]. However, the postprocessing compatibility of other ferroelectric materials with CMOS processes still warrants exploration. This section summarizes recent research on hafnium-based CMOS-integrated ferroelectric memory systems.

**Fig. 13:**
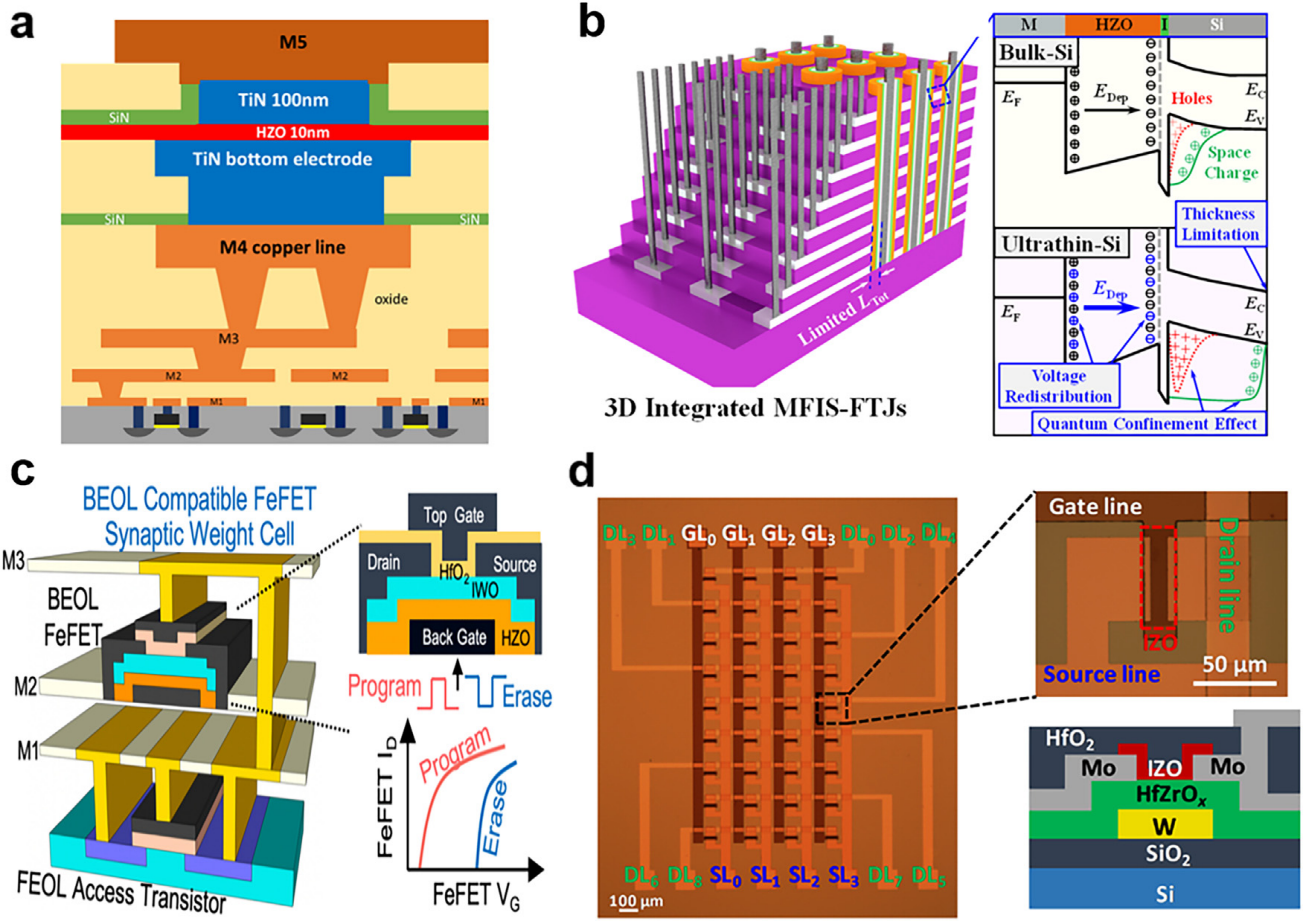
**CMOS integrated ferroelectric memory devices.** (a) FeRAM based on an HZO capacitor structure compatible with CMOS processing [117] (Copyright 2019 IEEE). (b) Schematic and band diagram of 3D integrated MFIS-FTJs [121] (Copyright 2022 IEEE). (c) Transistors consisting of FEOL-compatible Si-NMOS and BEOL-compatible HZO-FeFET and enabling monolithic 3D integration [122] (Copyright 2020 IEEE). (d) Optical image (left) and structural sketch (right) of an FeTFT composed of IZO and HfZrO_x_ in the array [91] (Copyright 2022 American Association for the Advancement of Science).

Scholars have recently studied hafnium-based ferroelectric capacitors, FTJs, and FeFETs with CMOS process compatibility [10,53,91]. Some scholars have also realized monolithic 3D integration on this basis [10,117]. Francois et al. [117] demonstrated the scalability of TiN/HZO/TiN (metal–ferroelectric–metal) ferroelectric capacitors integrated with CMOS technology in a 130-nm BEOL process with an approximately 16-kbit 1T1C array (Fig. 13a). These scaled bit cells were observed to have excellent performance (remnant polarization *P*_r_ > 40 µC/cm^2^, endurance > 10^11^ cycles, switching time < 100 ns, and operating voltage < 4 V). In addition, Liu et al. [118] presented hafnium-based optoelectronic memcapacitors that can achieve photoelectric perception and storage; they reported these devices to exhibit a capacitance memory window of 5.0 fF/µm^2^, a high/low capacitance ratio of 41, a retention time of 4 × 10^9^ s, and endurance of 10^9^ cycles. They also demonstrated a metal–insulator–semiconductor ferroelectric capacitor based on a HfAlO_x_ thin film and with non-volatile programmable capacitance [119,120]. These technological and structural breakthroughs have paved the way for CMOS-compatible hafnium-based ferroelectric memory in neuromorphic computing. A recent study proposed another method for stacking FTJs, namely the metal–ferroelectric–insulator–semiconductor (MFIS) FTJ with ultrathin semiconductor electrode technology based on HZO (Fig. 13b) [121]. This device, with an 8.5-nm-thick semiconductor electrode, was discovered to achieve an approximately 40 times better TER effect compared with that in bulk reference devices.

Regarding HfO_2_-based FeFETs, Dutta et al. [122] presented a transistor consisting of a front-end-of-line (FEOL)-compatible Si–N-type metal–oxide–semiconductor (NMOS) and BEOL-compatible HZO-FeFET that enables monolithic 3D integration (Fig. 13c). They integrated HZO with amorphous In_2_O_3_ doped with 1% tungsten (IWO) by using a low-thermal-budget process. The IWO FeFET with an ultrashort channel length of 20 nm achieved a 0.45 V read memory window, 100 ns write time, and >10^8^ endurance cycles. Dutta et al. [122] also reported 2-bit/cell synaptic weight cells. They used the VGG-8 model to make inferences from the Canadian Institute for Advanced Research (CIFAR)−10 image dataset; their compute-in-memory (CIM) system-level analysis demonstrated that the energy efficiency of the 22-nm-BEOL FeFET with a small array area was three times higher than that of 7-nm-static RAM. Fig. 13d presents another ferroelectric synaptic array-based CMOS-compatible CIM accelerator. The array unit is based on a composite of indium zinc oxide (IZO) and HfZrO_x_. The conductance of the FeTFT synapses can be linearly modulated by adjusting the ferroelectric polarization state. The array can also be used to extract key features from input images [91].

## 6 Conclusion and outlook

In-memory computing architectures offer an excellent solution to the problem of increasing data communication costs inherent in the conventional von Neumann architecture. Such architectures can also overcome the limitations engendered by the memory wall problem and can essentially eliminate the data movement delays and high-power consumption inherent in traditional computing architectures. Ferroelectric materials have become strong candidates for realizing brain-like synaptic devices because they are non-volatile and their polarization state can be precisely controlled. Neuromorphic devices based on ferroelectric materials and ANNs trained with ferroelectric synaptic devices have been recent hotspots of research, and fruitful results have been obtained [5,10,81]. Scholars have proposed numerous ferroelectric solid-state synaptic devices with low power consumption, high stability, high repeatability, and high controllability. Effective systems with functions such as handwritten digit recognition, graphic classification, waveform classification, edge detection, and time-series signal processing can be achieved using the crossbar array approach and by training ANNs [68,93,98,109].

Despite these notable advances in ferroelectric memory, some problems warrant further consideration. For example, factors affecting the resistive switching behavior of FTJs include ion migration, interfacial effects, and oxygen vacancies. Of these, the reversible migration of oxygen vacancies has a critical effect in oxide-based FTJs [123]. During one operating cycle, charge-trapping sites caused by defects at the interface of and within ferroelectric films gradually accumulate and severely affect the switching behavior of the device. This is a major problem that must be urgently solved if FTJ-based neural networks are to be practical.

Ferroelectric memory transistors are generally limited by the imprint effect and retention problems. The imprint effect is a phenomenon in which the ferroelectric polarization is more inclined in a specific direction; that is, one polarization state is more stable than the other. Thus, a stronger electric field is required to achieve reversal in one polarization direction, with only a weak field being required for reversal in the other polarization direction. In a hysteresis loop, this effect appears as an overall shift to the left or right and results in unequal positive and negative coercive fields [64,124]. Retention problems occur when the polarization strength gradually decreases over time. The main causes of such problems include spontaneous polarization decay caused by a depolarization field and polarization charge compensation caused by leakage current [16]. The presence of a depolarization field increases the electrostatic energy of a ferroelectric material. If the bound charges are not well shielded by the electrodes, the strong built-in electric field leads to an unstable uniform polarization state. This problem severely affects the lifetime of ferroelectric materials and their use in non-volatile synaptic applications.

FeS-FET devices can effectively overcome the limitations of memory threshold and storage state shifts in traditional transistors. In addition, a naturally existing movable charge forms its own electric field, effectively shielding the depolarization field in the device. This makes traditional FeFETs more durable and avoids the effects of charge trapping and leakage current, resulting in optimal ferroelectric memory device performance [64]. Notably, the time-dependent imprint effect and depolarization phenomena that occur in ferroelectric memory devices can be utilized for temporal neuromorphic computing tasks, such as in dynamic analysis and prediction tasks in RC neural networks.

In-sensor computing technology that integrates sensing, computing, and storage functions has become another new technological growth point after in-memory computing technology. However, in-sensor computing technology based on ferroelectric materials remains in its infancy, and many branches remain to be explored and investigated. Most of the existing devices simply integrate only perception and storage functions or integrate perception and storage functions along with a simple processing stage. Practical in-sensor computing is still in its infancy.

On the basis of the findings of this review, we propose three considerations that are not limited to ferroelectric materials. First, in terms of device performance, most of the existing in-sensor computing devices are based on the simple processing of only a single sense, such as vision and touch. Therefore, for such device, processing power has considerable room for improvement. However, actual application scenarios are extremely complex and affected by many factors. Ferroelectric materials have various properties—such as piezoelectric, optoelectronic, and pyroelectric properties—ferroelectric synaptic devices coupled with more than two stimuli responses should be considered for future multiple in-sensor computing applications. Therefore, developing a device system with multisense fusion and diverse processing functions is essential, and this is the main direction of future development of brain-like devices. Furthermore, developing a general system for solving the problem of coupling among multiple sensors and for realizing multifunctional and energy-efficient sensing is imperative. Second, in terms of array integration, the processing capability of small-scale arrays is limited and does not have much room for improvement. Therefore, solving the problems faced in integration technology and developing 3D integration technology are essential for in-sensor computing technology. Finally, the information received externally is only simply preprocessed by the current in-sensor computing devices or systems. Subsequently, the peripheral control circuits are required to help to transfer the information to more complex information-processing environments to solve practical problems. Research in this area is still in its infancy. Hence, further in-depth research is warranted on information-processing architectures, task scheduling, division of labor, and other strategies related to in-sensor computing.

## Declaration of competing interest

The authors declare that they have no conflicts of interest in this work.
